# Glucocorticoid-glucocorticoid receptor-HCN1 channels reduce neuronal excitability in dorsal hippocampal CA1 neurons

**DOI:** 10.1038/s41380-022-01682-9

**Published:** 2022-07-15

**Authors:** Jiwon Kim, Yun Lei, Xin-Yun Lu, Chung Sub Kim

**Affiliations:** Department of Neuroscience & Regenerative Medicine, Medical College of Georgia at Augusta University, Augusta, GA 30912 USA

**Keywords:** Neuroscience, Physiology, Psychology

## Abstract

While chronic stress increases hyperpolarization-activated current (*I*_h_) in dorsal hippocampal CA1 neurons, the underlying molecular mechanisms are entirely unknown. Following chronic social defeat stress (CSDS), susceptible mice displayed social avoidance and impaired spatial working memory, which were linked to decreased neuronal excitability, increased perisomatic hyperpolarization-activated cyclic nucleotide-gated (HCN) 1 protein expression, and elevated *I*_h_ in dorsal but not ventral CA1 neurons. In control mice, bath application of corticosterone reduced neuronal excitability, increased tetratricopeptide repeat–containing Rab8b-interacting protein (TRIP8b) and HCN1 protein expression, and elevated *I*_h_ in dorsal but not ventral CA1 region/neurons. Corticosterone-induced upregulation of functional *I*_h_ was mediated by the glucocorticoid receptor (GR), HCN channels, and the protein kinase A (PKA) but not the calcium/calmodulin-dependent protein kinase II (CaMKII) pathway. Three months after the end of CSDS, susceptible mice displayed persistent social avoidance when exposed to a novel aggressor. The sustained behavioral deficit was associated with lower neuronal excitability and higher functional *I*_h_ in dorsal CA1 neurons, both of which were unaffected by corticosterone treatment. Our findings show that corticosterone treatment mimics the pathophysiological effects of dorsal CA1 neurons/region found in susceptible mice. The aberrant expression of HCN1 protein along the somatodendritic axis of the dorsal hippocampal CA1 region might be the molecular mechanism driving susceptibility to social avoidance.

## Introduction

Post-traumatic stress disorder (PTSD) is a debilitating long-term mental disease that can develop following exposure to traumatic, life-threatening events such as physical, emotional, or sexual attacks, natural disasters, wars, and pandemics [1]. Reliving the horrific incident, actively avoiding unpleasant memories or cues, negative mood and thoughts, such as emotional numbing and anhedonia, and hyperarousal, such as irritability, anger, hypervigilance, and sleep issues, are all symptoms of PTSD [1]. Individuals, families, and society face significant issues in terms of well-being, relationships, and financial burden due to these complex symptoms [2–4]. Additionally, those with PTSD are more likely to have comorbid conditions such as depression, anxiety, cardiovascular disease, and drug abuse disorder [5, 6], which increases the chance of attempting suicide [7, 8]. One of the most common symptoms of PTSD is avoidance. Experiential avoidance is defined as attempts to avoid private traumatic experiences (i.e., thoughts, feelings, memories, physical sensations), even if doing so causes long-term harm [9]. A growing body of literature suggests that experiential avoidance is the key diagnostic feature of PTSD [10–13].

Animal models are critical for researching the pathophysiology of PTSD because they allow researchers to track the development of PTSD over time using controlled stresses. It is worth noting that it only affects a small percentage of persons who have been exposed to potentially fatal events. Chronic social defeat stress (CSDS) is a psychosocial stress animal model that causes PTSD symptoms like social avoidance, emotional shifts, and anxiety-like behavior [14]. CSDS produces interindividual variation (i.e., susceptible and resilient phenotypes) [14], similar to that seen in patients with PTSD. This animal model is a valuable resource for understanding the mechanisms underlying the development of susceptibility and resilience to social avoidance.

Functional neuroimaging of PTSD patients reveals structural and functional changes in the hippocampus, amygdala, and medial prefrontal cortex [15–17]. In PTSD patients [18–20], researchers found that decreased hippocampal volume, hypoactivity of the hippocampus, and disruption of the hypothalamic–pituitary–adrenal (HPA) axis. The hippocampus is a critical brain region in PTSD symptoms such as spatial working memory and emotion-related behavior [18, 21]. The hippocampus, which includes high amounts of mineralocorticoid receptor and GR, regulates HPA-axis activity among limbic brain structures [22]. Cortisol levels in PTSD patients are higher than in the healthy control group when they re-experience stressful situations [23–25]. Administration of exogenous glucocorticoids has been linked to decreased memory performance in healthy people [26, 27]. Animals treated with corticosterone or repeated stress impair spatial working memory [28, 29]. The hippocampus of rodents can be separated anatomically and functionally into two brain regions: the dorsal hippocampus, which corresponds to the human posterior hippocampus, and the ventral hippocampus, which corresponds to the human anterior hippocampus [30, 31]. The dorsal hippocampus is mainly linked to spatial learning and memory, while the ventral hippocampus is associated with emotion-related reactions like anxiety [31–33]. Furthermore, dorsal and ventral CA1 regions of the hippocampi have distinct electrophysiological properties. Compared to ventral CA1 neurons, dorsal CA1 neurons have a hyperpolarized resting membrane potential, lower input resistance, and reduced neuronal excitability [34].

HCN channels generate membrane currents that are characterized by (1) cyclic nucleotide-mediated modulation, (2) Na^+^ and K^+^ permeability, and (3) activation by membrane hyperpolarization [35]. HCN1 is the major isoform expressed in the hippocampus, neocortex, and cerebellar cortex, although there are four isoforms of HCN channels (HCN1–HCN4) [36, 37]. In the hippocampal CA1 region, HCN1 expression displays a gradient of increasing channel density along the somatodendritic region of CA1 [38, 39]. A portion of HCN channels is active at resting membrane potential, resulting in a non-inactivating inward current that affects intrinsic membrane properties, neuronal excitability, synaptic integration, and synaptic plasticity [40]. Evidence suggests a link between HCN channels and mental diseases, including depression and anxiety [41–46]. Whole-brain knockout mice lacking either the pore-forming protein (HCN1 and HCN2) or an auxiliary channel subunit, tetratricopeptide repeat–containing Rab8b-interacting protein (TRIP8b), exhibit antidepressant-like behaviors [41]. Normal rats demonstrate antidepressant- and anxiolytic-like effects when HCN1 protein is lowered in the dorsal but not ventral CA1 area [42]. Rats exposed to chronic unpredictable stress (CUS) for two to four weeks show depressive- and anxiogenic-like behaviors associated with the upregulation of perisomatic *I*_h_ in the dorsal but not ventral CA1 region/neurons [43, 44]. These findings imply that chronic stress, but not acute stress, causes pathogenic alterations in CA1 neurons in the dorsal but not ventral hippocampi. The molecular mechanisms driving pathogenic changes in *I*_h_, on the other hand, are unknown.

This study found that CSDS produced susceptible and resilient phenotypes during the social interaction test. Although both susceptible and resilient mice showed anxiogenic-like behavior during the elevated plus-maze test, spatial working memory was impaired in susceptible mice compared to control and resilient mice in the Y maze spontaneous alternation test. Compared to control and resilient mice, dorsal but not ventral CA1 neurons had lowered input resistance (R_in_) and reduced neuronal excitability. In susceptible mice, perisomatic HCN1 protein expression and *I*_h_ were elevated in the dorsal but not ventral CA1 neurons/region, indicating increased functional *I*_h_ (i.e., decreased R_in_ and neuronal excitability). In control mice, bath application of corticosterone reduced R_in_ and decreased neuronal excitability in the dorsal but not ventral CA1 neurons. We further found that corticosterone pretreatment increased *I*_h_ as well as TRIP8b and HCN1 protein expression in the perisomatic CA1 region/neurons of the dorsal hippocampus. Corticosterone-induced functional *I*_h_ was mediated by the GR, HCN channels, and the PKA but not the CaMKII pathway. Susceptible mice were reintroduced to novel aggressor mice one month or three months after the end of the CSDS and displayed persistent social avoidance. This long-term behavioral impairment was linked to decreased neuronal excitability, increased TRIP8b and HCN1 protein expression, and elevated *I*_h_ in the dorsal CA1 region/neurons. On the other hand, resilient mice displayed increased neuronal excitability in the dorsal CA1 neurons. Corticosterone had no additional impacts on functional *I*_h_ in the dorsal CA1 neurons from susceptible mice, but it did in control and resilient mice. Our findings show that functional *I*_h_ may be involved in cellular pathways that drive the development of susceptibility and resilience to social avoidance.

## Materials and methods

### Animals

Male wild-type C57BL/6 J mice (7 to 16 weeks old) were used in this study. Mice were housed 5 per cage on a 12 h light schedule (on 7:00 A.M. off 7:00 P.M.) with ad libitum access to water and food. In a study with one month or three months without CSDS, mice were single-housed following CSDS. All procedures involving animals were approved by the Institutional Animal Care and Use Committee of Augusta University.

### Drugs

Corticosterone (Cat # 3685), dexamethasone(Cat # 1126), mifepristone (RU 486; Cat # 1479), KT5720 (Cat #1288), KN-62 (Cat #1277), and ZD7288 (Cat #1000) were purchased from Tocris. Corticosterone, Dexamethasone, KT5720, and KN-62 were dissolved in dimethyl sulfoxide and diluted to a final concentration in artificial cerebral spinal fluid (aCSF).

### Chronic social defeat stress

Social defeat was generated using a resident–intruder paradigm with minor modifications [47]. 3-to-4-month-old male CD-1 mouse was co-housed with ovariectomized female CD-1 mouse for at least two weeks. Subsequently, male sexually experienced CD-1 mice (residents) were housed individually for one week before being screened for aggression. During the screening process, a ‘screener’ C57BL/6 J mouse was introduced into the cage of a singly housed CD-1 mouse for 3 min/session/day and the latency of the CD-1 mouse to attack the ‘screener’ C57BL/6 J mouse during each session was recorded. The screening procedure was repeated for three consecutive days. The male CD-1 mice with the attack latency <60 s for at least two sessions on the last day were selected for aggressors in social defeat experiments. For the chronic social defeat procedure, male C57BL/6 J experimental mice were individually introduced into the large home cage of an aggressor CD-1 mouse for 5 to 15 min during which time the experimental mouse was physically and emotionally defeated. After 5 to 15 min aggression encounter session, the CD-1 aggressor mouse and the experimental C57BL/6 J mouse were housed together but separated by a perforated Plexiglass divider to allow sensory contact for the remainder of the 24-h period. The following day, the experimental C57BL/6 J mice were re-exposed to a new CD-1 aggressor mouse for 5–15 min of direct physical contact and then a 24-h period of emotional contact. The procedure was repeated for 12 days total. Control C57BL/6 J mice were housed two per cage in the cages identical to those used for socially defeated mice. After the last social defeat stress, control and defeated mice were individually housed in standard cage. Selection of aggressors and social defeat stress were performed between 4 pm and 6 pm.

### Behavioral experiment

All behavioral measurements were analyzed by custom-written programs. All behavioral experiments were performed between 4 pm and 6 pm. All experiments were performed under blind conditions.

### Social interaction test

Social interaction test was assessed at 48 h after the last social defeat stress session. Mice were acclimated to a behavior room under red-light conditions (~4 lux) for at least 1 h. The apparatus is made of a box (18 × 18 × 12 in) with a perforated Plexiglass cage (4 × 4 × 12 in) at the middle of one side of the box. The social interaction test consisted of a two-trial procedure under red-light conditions (~4 lux). The experimental mice were placed in the corner of the open arena and allowed to freely explore for 2.5 min (i.e., 1st session without social target). After the 1st session, an unfamiliar CD1 male mouse was introduced into the perforated Plexiglass cage as a social stimulus for 2.5 additional min (i.e., 2nd session with social target). The surface of the open field was cleaned with 70% EtOH in order to remove permeated odors by previous animals after each trial. A CCD camera placed above the apparatus recorded behavior. A social interaction ratio was calculated as the ratio of the time spent in the interaction zone in the presence of a social target to the time spent in the interaction zone without a social target. Mice with scores more than 1 were defined as “resilient” and those with scores less than 1 were defined as “susceptible” [47].

### Elevated plus-maze test

Anxiety was tested on the elevated plus-maze apparatus described previously [43]. The apparatus is made of a black acrylic sheet. Four arms (12 in long and 3 in wide) are connected and elevated to a height of 16 in from the floor. Mice were placed on the central platform facing an open arm and allowed to explore the maze for 6 min. The surface was cleaned with 70% EtOH to remove permeated odors by previous animals after each trial. Behavior in the elevated plus maze was recorded for 6 min using a CCD camera. The time spent in the open arm and the number of total arm entries were determined. Arm entry was defined as 50% of the body being positioned within the arm.

### Y maze spontaneous alternation test

Short-term spatial working memory was examined using the Y maze spontaneous alternation test as previously described [28]. The apparatus consists of three identical arms (each arm: 14 in x 3 in; no intra-maze cues) diverging at 120^o^ angle from each other. In the Y maze, there is no blocking off of each arm. The mice were placed in the one arm of the Y maze (start arm) and allowed to freely explore the arena for 6 min. The sequence of arm entries and the total number of arm entries were measured. The surface of the maze was cleaned with 70% EtOH in order to remove permeated odors by previous animals after each trial. A CCD camera placed above the apparatus recorded behavior. Arm entry is defined as having all four paws in the arm. An alternation is defined as successive entry into three different arms, on overlapping triplet sets. The percentage of alternations was calculated as the number of actual alternations divided by the maximum number of alternations (the total number of arm entries minus 2).

### Acute hippocampal slice preparation

Mice were anesthetized with a lethal dose of isoflurane (>5%) in a closed plastic container and transcardially perfused with ice-cold aCSF composed of (in mM): 2.5 KCl, 1.25 NaH_2_PO_4_, 25 NaHCO_3_, 0.5 CaCl_2_, 7 MgCl_2_, 7 dextrose, 210 sucrose, 1.3 ascorbic acid, and 3 sodium pyruvate, bubbled with 95% O_2_ − 5% CO_2_. The brain was removed and hemisected along the longitudinal fissure. Dorsal (coronal section with an angle of 10–20°) and ventral (horizontal section) hippocampal slices were prepared as previously described [43]. Hippocampal slices (300 μm thick) were made in ice-cold aCSF using a vibrating microtome (Microslicer DTK-Zero1, DSK, Kyoto, Japan). Slices were placed in a holding chamber containing (in mM) 125 NaCl, 2.5 KCl, 1.25 NaH_2_PO_4_, 25 NaHCO_3_, 2 CaCl_2_, 2 MgCl_2_, 12.5 dextrose, 1.3 ascorbic acid, and 3 sodium pyruvate, bubbled with 95% O_2_ − 5% CO_2_ at 35 °C for 30 min and then incubated for at least 45 min at room temperature before used for electrophysiology.

### Whole-cell patch-clamp recordings

Whole-cell current-clamp recordings were performed as previously described [34, 42, 43]. Briefly, hippocampal slices were submerged in a recording chamber continuously perfused with aCSF containing (in mM) 125 NaCl, 3 KCl, 1.25 NaH_2_PO_4_, 25 NaHCO_3_, 2 CaCl_2_, 1 MgCl_2_, and 12.5 dextrose, bubbled with 95% O_2_ − 5% CO_2_ at a rate of 1 ml/min and 31–33 °C. For whole-cell voltage-clamp recordings, dorsal hippocampal slices were transferred to the recording chamber and perfused with aCSF containing (in mM) 125 NaCl, 3 KCl, 1.25 NaH_2_PO_4_, 25 NaHCO_3_, 2 CaCl_2_, 1 MgCl_2_, and 12.5 dextrose, 1 BaCl_2_, 10 TEA-Cl, 0.2 3,4-DAP bubbled with 95% O_2_ − 5% CO_2_ at a rate of 1 ml/min and 31–33 °C. CA1 pyramidal neurons were visually identified using a microscope (Olympus BX51WI, US) fitted with differential interference contrast optics [48]. Patch pipettes for somatic (4–7 MΩ) were prepared with capillary glass (external diameter 1.65 mm and internal diameter 1.1 mm, World Precision Instruments) using a Flaming/Brown micropipette puller (P-1000, Sutter Instrument, CA) and filled with an internal solution containing (in mM) 120 K-gluconate, 20 KCl, 10 HEPES, 4 NaCl, 7 K2-phosphocreatine, 4 Mg-ATP, 0.3 Na-GTP (pH 7.3 with KOH). Whole-cell recordings were performed using a MultiClamp 700B amplifier (Molecular Devices, LLC., CA) and commercial acquisition software pCLAMP10 (Molecular Devices, LLC., CA). Electrical signals were filtered at 10 kHz, sampled at 20 kHz, and digitized by Axon Digidata 1440 A (Axon Instruments). For whole-cell current-clamp recordings, series resistance was monitored during each experiment and experiments were discarded if the series resistance exceeded 30 MΩ for somatic recordings. The resting membrane potential was the potential of the soma in the absence of any injected current. Liquid junction potential was not corrected and was estimated to be about −13 mV using the Patcher’s Power Tools add-on in Igor Pro.

### Cell-attached patch-clamp recordings

Cell-attached voltage-clamp recordings were performed as previously described [43, 44]. Pipettes contained the following (in mM): 120 KCl, 10 HEPES, 2.0 CaCl_2_, 1.0 MgCl_2_, 20 TEA-Cl, 0.2 3,4-DAP, 1 BaCl_2_, pH 7.3 with KOH. Membrane currents were recorded using a MultiClamp 700B amplifier (Molecular Devices, LLC., CA) and commercial acquisition software pCLAMP10 (Molecular Devices, LLC., CA). Electrical signals were sampled at 10 kHz, analog filtered at 2 kHz by using Axon Digidata 1440 A (Axon Instruments). Maximal *I*_h_ was measured by using hyperpolarizing voltage commands to −140 mV from a holding potential of −30 mV. Linear leakage and capacitive currents were digitally subtracted by scaling traces at smaller command voltages in which no voltage-dependent current was activated.

### DAB staining

Neuronal reconstructions were performed as previously described [43]. Briefly, slices containing neurobiotin-filled neurons were fixed in a 3% glutaraldehyde solution (0.1 M phosphate buffer, pH 7.4) and stored at 4 °C before undergoing histological processing using an avidin-HRP system activated by diaminobenzidine (DAB; Vector Laboratories). DAB-processed slices were mounted in glycerol and viewed with a microscope (Revolve, ECHO). Images of the cell were created from z-stacks of photos taken at 10X under a microscope. The location of patched CA1 neurons in the dorsal and ventral hippocampus were determined. Experiments were performed under blind conditions.

### Immunohistochemistry

Immunohistochemistry was carried out as described previously [42, 43]. 300 μm thick slices were further sectioned using a freezing microtome into 50-μm thin sections and stored in cryoprotectant (30% sucrose, 30% ethylene glycol, 1% polyvinyl pyrrolidone, 0.05 M sodium phosphate buffer). Sections were briefly rinsed in PBS buffer and incubated in 0.1% TritonX-100 for 30 min. Subsequently, slices were blocked in PBS solution containing 5% normal goat serum, 0.03% TritonX-100 for 1 h, and then incubated in primary antibody diluted in blocking solution overnight at 4 °C. Slices were rinsed in PBS buffer and then incubated in secondary antibody for 1 h at room temperature. Primary antibody in this study was used as follows; rabbit-anti-HCN1 (1:500, Invitrogen, Cat # PA5-78675), rabbit anti-HCN2 (1:500, Invitrogen, Cat # PA1-918), rabbit anti-HCN3 (1:500, Invitrogen, Cat # PA5-104434), rabbit anti-HCN4 (1:500, Invitrogen, Cat # PA5-111793), rabbit C-terminal TRIP8b (1:500, Proteintech, Cat #13084-1-AP), and rabbit anti-GR (1:500, Invitrogen, Cat # PA1-511A). Experiments were performed under blind conditions.

### Western blotting

Western blotting was carried out as previously described [42, 43]. After hippocampal acute slices, the dorsal CA1 region was isolated using 26-gague needles under a dissecting microscope on ice. Isolated dorsal CA1 tissue was stored at −80 °C until processing for western blotting. The protein was separated on 10% sodium dodecyl sulfate-polyacrylamide gel and transferred to nitrocellulose membrane (Invitrogen). The membrane was incubated with blocking solution (LI-COR) for 1 h at room temperature and then with the primary antibody (rabbit anti-HCN1 1:500, Invitrogen; rabbit anti-GR 1:500, Invitrogen; mouse anti- β-tubulin 1:2000, Sigma-Aldrich) overnight at 4 °C. The membrane was rinsed in PBST buffer (0.05% Tween 20) 3 × 10 min, and then incubated in secondary antibody (Li-COR) for 1 h at room temperature. Experiments were performed under blind conditions.

### Data analysis

Input resistance was measured by the slope of the linear fit of the V-I plot between +30 and −150 pA current injections. Single action potentials (APs) were analyzed for AP threshold, AP amplitude, AP half-width, and maximum *dv/dt* (mV/msec). AP threshold was defined as the voltage where dv/dt of the spike crossed 20 mV/ms. AP amplitude was determined from threshold to peak, with the half-width measured at half this distance.

### Statistical analysis

Statistical comparisons were performed using ANOVA (one-factor or two-factor) followed by Bonferroni post-hoc test, paired *t*-test (Wilcoxon signed-rank test) or unpaired *t*-test (Mann-Whitney *U* test) with GraphPad software. The results of all statistical analyses were compiled into an word file. *P < 0.05 was considered as statistically significant.

## Results

### Susceptible mice displayed social avoidance and impaired spatial working memory

**Fig. 1:**
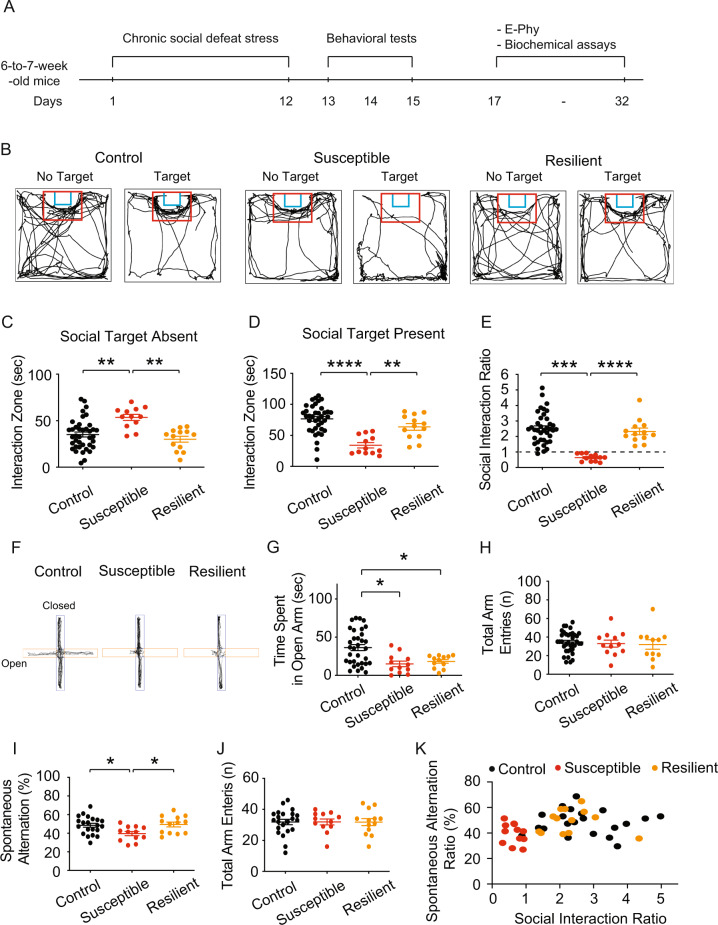
Susceptible mice displayed social avoidance and impaired spatial working memory. **A** Timeline of chronic social defeat stress, behavioral tests, electrophysiology, and biochemical assay. **B** Representative video tracking images of control, susceptible, and resilient mice without and with a social target during the social interaction test. **C** In the absence of the social target, susceptible group spent more time in the interaction zone than the control and resilient groups. **D** In the presence of a social target, susceptible group spent less time in the interaction zone than the control and resilient groups. **E** During the social interaction test, chronic social defeat stress resulted in the formation of susceptible and resilient phenotypes. **F** Representative video tracking images of age-matched mice during a 6-min elevated plus maze test. **G**, **H** The susceptible and resilient groups spent less time in the open arm than the control group without changing the total arm entries. **I**, **J** The percentage of spontaneous alternation in susceptible group was much lower than in the control and resilient groups, despite no differences in total arm entries. **K** The relationship between social avoidance and spatial working memory between groups. Data are expressed as mean ± SEM.

We used male mice aged 6 to 7 weeks and subjected them to CSDS for 12 days. (Figs. 1A and S1A). Mice were tested in the social interaction test, which assesses social avoidance, to see if CSDS affected social behavior [49]. When CSDS-treated mice were not exposed to a novel conspecific, they spent a similar amount of time in the interaction zone as the control group. (Fig. S1C). CSDS-treated mice spent less time in the interaction zone than control mice when a new conspecific was brought into the cage as a social stimulus (Fig. S1D). Based on their social interaction ratio (Fig. S1F), CSDS-treated mice were separated into susceptible and resilient subgroups [14]. Susceptible mice spent more time in the interaction zone than control and resilient mice in the absence of a novel conspecific (Fig. 1B, C). In the presence of a novel conspecific, susceptible mice spent less time in the interaction zone than control and resilient mice (Fig. 1B, D). We found that CSDS could produce both susceptible and resilient phenotypes (Fig. 1E). The anxiety levels of the control and CSDS-treated groups were subsequently assessed using an elevated plus-maze test (Fig. 1A) [50]. Both susceptible and resilient mice spent less time in the open arm than the control group, but there was no difference in the overall number of arm entries (Fig. 1F, G). Hippocampal dysfunction is a crucial contributor to PTSD symptoms such as working memory [51, 52]. We further evaluate the spatial working memory of these animals using the Y maze spontaneous alternation test (Y maze test). The Y maze test is a frequently used method for assessing spatial learning and memory in mice by monitoring their propensity to explore novel surroundings [53]. In comparison to the control and resilient mice, susceptible mice demonstrated a lower percentage of spontaneous alternation (Fig. 1I) but no differences in total arm entries (Fig. 1J). The relationship between social avoidance and spatial working memory can be distinguished between susceptible and control or resilient mice (Fig. 1K).

### Susceptible mice had a decrease in neuronal excitability of dorsal but not ventral CA1 neurons

**Fig. 2:**
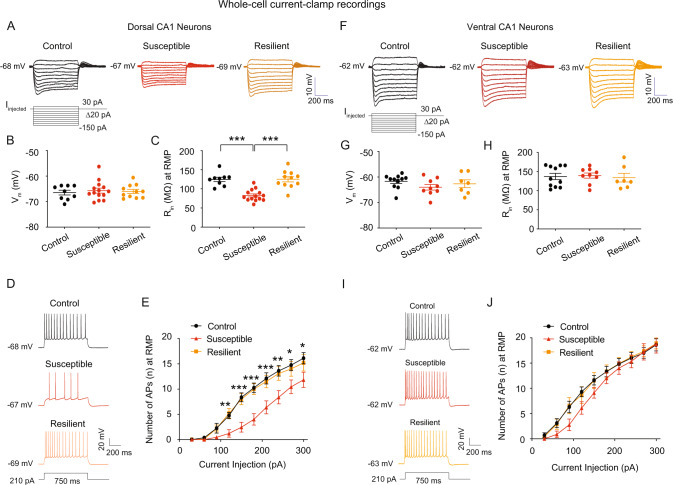
The susceptible group reduced R_in_ and neuronal excitability in dorsal CA1 neurons, but not in ventral CA1 neurons. **A**–**J** We performed whole-cell current-clamp recordings. **A** representative voltage responses with step current commands ranging from −150  pA to +30 pA (Δ = 20 pA) at RMP. **B** There was no difference in RMP of dorsal CA1 neurons between groups. **C** The dorsal CA1 neurons of the susceptible group had lower R_in_ at RMP than the control and resilient groups. **D** Representative voltage responses with depolarizing current step (210 pA; 750 ms) at RMP in dorsal CA1 neurons from the control, susceptible, and resilient groups. **E** Dorsal CA1 neurons from susceptible group had lower action potential firing than the control and resilient groups. **F** Representative voltage responses with step current commands ranging from −150 pA to +30 pA (Δ = 20 pA) at RMP. **G**, **H** There were no differences in RMP and R_in_ at RMP of ventral CA1 neurons between groups. **I** Representative voltage responses with depolarizing current step (210 pA; 750 ms) at RMP in the ventral CA1 neurons from the control, susceptible, and resilient groups. **J** Action potential firing of ventral CA1 neurons was not different between groups. Data are expressed as mean ± SEM.

Since susceptible mice displayed social avoidance and impaired spatial working memory, we used somatic whole-cell current-clamp recordings to see if the electrophysiological properties of hippocampal CA1 neurons were changed. Acute dorsal and ventral hippocampal slices were prepared after behavioral tests [43]. We put aside tissue samples for western blotting (i.e., cut the CA1 region) and immunohistochemistry after acute hippocampal slices. As a result, we can correlate electrophysiological data with protein expression. The resting membrane potential of dorsal CA1 neurons did not differ across groups (Fig. 2A, B). R_in_ of dorsal CA1 neurons was considerably lower in susceptible mice compared to the control and resilient mice at resting membrane potential (RMP) (Fig. 2C) and at −65 mV (Fig. S2A, B). The number of action potentials at RMP in dorsal CA1 neurons from susceptible mice was significantly lower than in the control and resilient mice (Fig. 2D, E), implying a reduction in neuronal excitability. We observed increased afterhyperpolarization from trains of action potentials of susceptible-dorsal CA1 neurons (data not shown), consistent with a reduction in neuronal excitability (Fig. 2D, E). RMP (Fig. 2F, G), R_in_ at RMP (Fig. 2F, H), R_in_ at −65 mV (Fig. S2C, D), and the number of action potentials at RMP (Fig. 2I, J), on the other hand, were not different across groups in ventral CA1 neurons. Because the number of action potentials at RMP was reduced in dorsal CA1 neurons from susceptible mice compared to the control and resilient mice, we examined the properties of a single action potential. In dorsal and ventral CA1 neurons, there were no differences in action potential threshold, half-width, amplitude, and max dv/dt between control, susceptible, and resilient groups (Supplementary Table 1). Since the magnitude of R_in_ is dependent on the longitudinal position of neurons in the CA1 region [54, 55], CA1 neurons were filled with neurobiotin during electrophysiological recordings and fixed for post-hoc morphological examination. There were no significant differences in the positions of recorded dorsal CA1 (Fig. S3A–C) and ventral CA1 (Fig. S3D–F) neurons across groups.

### Susceptible mice had increased HCN1 protein expression and elevated *I*_h_ in dorsal CA1 region/neurons

**Fig. 3:**
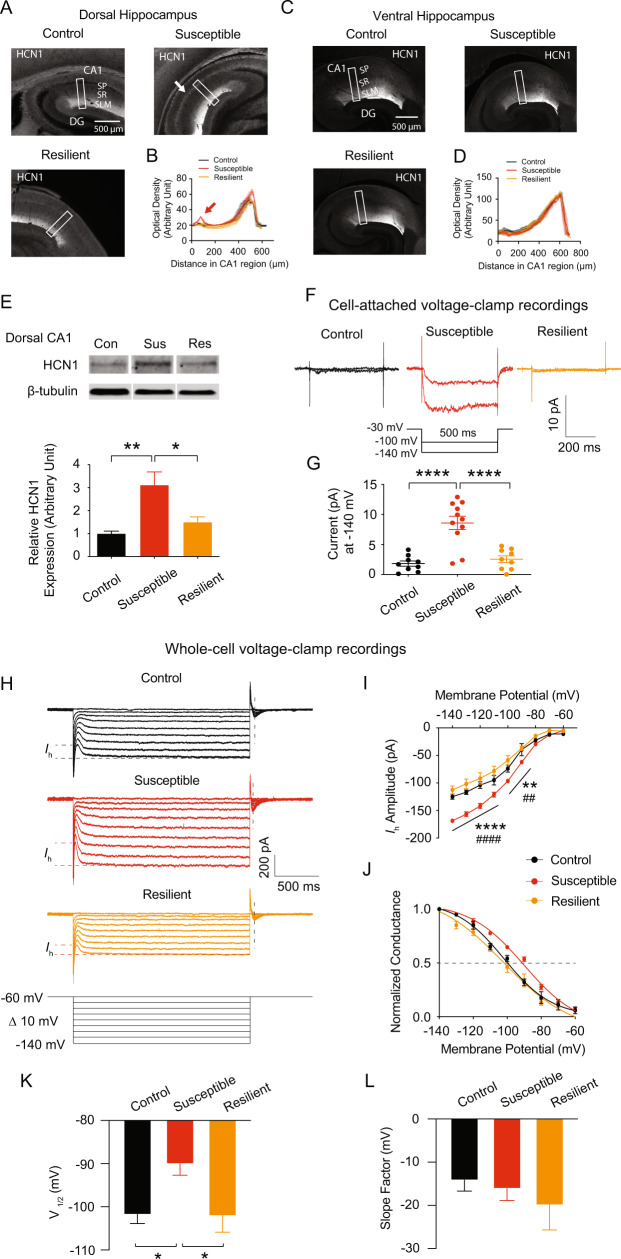
HCN1 protein expression and Ih were significantly increased in dorsal CA1 region/neurons from susceptible group. **A**, **C** Representative dorsal and ventral hippocampal slices immunolabeled with antibody against HCN1. Rectangle boxes depict the region of the slice used for quantification of the optical density. The arrows indicate increased perisomatic HCN1 protein expression. **B**, **D** Quantification of HCN1 protein expression from the perisomatic region to the distal dendritic region of CA1 from the dorsal and ventral hippocampi. **E** Western blot (top) and quantification (bottom) of HCN1 protein in dorsal CA1 region from the control, susceptible, and resilient groups. **F**, **G** We performed cell-attached voltage-clamp recordings. **F** Representative maximal *h* current traces in response to a 500-ms hyperpolarizing voltage step (−140 mV). **G** *I*_h_ was significantly increased in dorsal CA1 neurons from susceptible group compared with those from the control and resilient groups. **H**–**L** We performed whole-cell voltage-clamp recordings. **H** Representative current responses with step voltage commands ranging from −140 mV to − 60 mV (Δ = 10 mV) at a holding potential of −60 mV. The approximate position for determining the peak tail current is shown by black vertical dashed lines. **I** Susceptible group showed increased *I*_h_ in the dorsal CA1 neurons compared with those from the control and resilient groups. **J** The voltage dependence of activation for *h* channel was determined from tail currents (*I*_h_ / *I*_h max_). The activation curve was fitted with a Boltzmann function with the following values: control V_1/2_ = −101.8 mV, k = −14.13 mV, susceptible V_1/2_ = −90.02 mV, k = −16.09 mV, resilient V_1/2_ = −102.1 mV, k = −19.9 mV. **K** The half-activation voltage of *h* channel (V_1/2_) for susceptible group was significantly shifted to the right by around +10 mV, whereas **L** the slope factor was not different between groups. Data are expressed as mean ± SEM.

HCN channels at RMP play an important role in intrinsic membrane properties including R_in_ [34]. We examined to see if HCN protein was altered in the hippocampal CA1 region. We used a 50 μm thick re-section from 300 μm thick slices for immunohistochemistry. Indeed, HCN1 protein expression was considerably increased in the perisomatic CA1 region of the dorsal (Fig. 3A, B) but not ventral (Fig. 3C, D) hippocampus in susceptible but not control or resilient mice. Because other HCN channel subunits are also expressed in the hippocampus, we examined whether they were altered in susceptible mice. None of the HCN channel subunits, on the other hand, were different across the groups (Fig. S4A–L). Furthermore, HCN1 protein expression in dorsal CA1 region was significantly greater in susceptible mice compared to the control and resilient mice (Fig. 3E). We then measured *I*_h_ at the soma of dorsal CA1 neurons using cell-attached patch-clamp recordings. After subtracting linear leakage and capacitive currents by scaling traces at −40 mV, the *h* current exhibited a lack of time-dependent component of *I*_h_ in response to voltage, as shown in Fig. 2A, F voltage traces. Patches from susceptible mice exhibited significantly higher *I*_h_ than patches from the control and resilient mice (Fig. 3F, G), which was consistent with increased HCN1 protein expression. Consistent with the cell-attached recordings, whole-cell voltage-clamp recordings revealed that *I*_h_ was significantly increased in the dorsal CA1 neurons of susceptible group compared to control or resilient group (Fig. 3H, I). We further found that the half-activation voltage (V_1/2_) of the *h* channel activation curve for susceptible group was significantly shifted to the right by around +10 mV (V_1/2_ of control: −101.8 mV; susceptible: −90.02 mV; resilient: −102.1 mV, Fig. 3J, K), whereas the slope factor was not different (Fig. 3J, L) compared to control and resilient groups.

### Dorsal CA1 neurons showed greater response to corticosterone than ventral CA1 neurons

**Fig. 4:**
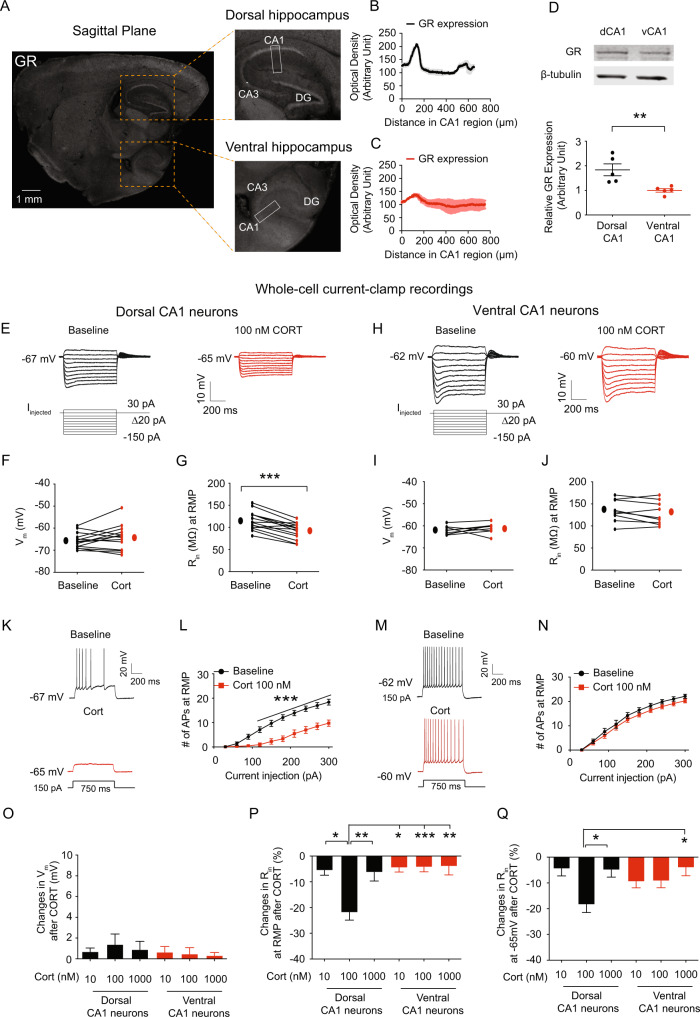
Dorsal CA1 neurons responded to corticosterone more strongly than ventral CA1 neurons. **A** Representative sagittal section of brain showing GR immunoreactivity in dorsal (top) and ventral (bottom) hippocampi. Rectangle boxes depict the region of the slice used for quantification of optical density. **B**, **C** Quantification of GR protein expression from the perisomatic region to the distal dendritic region of CA1 from the dorsal and ventral hippocampi. **D** Western blot (top) and quantification (bottom) of GR protein in CA1 region from the dorsal and ventral hippocampi. **E**–**Q** We performed whole-cell current-clamp recordings. **E**, **H** Representative voltage responses with step current commands ranging from −150 pA to +30 pA (Δ = 20 pA) at RMP before and after bath application of corticosterone. Corticosterone lowered R_in_ (**G**) but had no impact on RMP (**F**) in dorsal CA1 neurons. There are no changes in RMP (**I**) and R_in_ (**J**) in ventral CA1 neurons following corticosterone treatment. Representative voltage responses with depolarizing current step (150 pA; 750 ms) at RMP in dorsal (**K**) and ventral (**M**) CA1 neurons. **L** Dorsal CA1 neurons had decreased action potential firing at RMP following corticosterone treatment. **N** Action potential firing at RMP was not altered in ventral CA1 neurons following corticosterone treatment. **O** Corticosterone doses of 10 nM, 100 nM, and 1 μM had no effect on RMP of CA1 neurons in dorsal and ventral hippocampi. 100 nM corticosterone significantly reduced R_in_ at RMP (**P**) and at −65 mV (**Q**) in dorsal CA1 neurons compared to 10 nM and 1 μM corticosterone. When compared to 10 nM and 1 μM corticosterone, 100 nM corticosterone had no impact on R_in_ at RMP (**P**) and at −65 mV (**Q**) in ventral CA1 neurons. Data are expressed as mean ± SEM.

Dysregulation of the HPA axis is common in people with severe mental illness including PTSD [56, 57]. When glucocorticoid levels are high, GR-expressing neurons in the hippocampus [58] and the hypothalamus [59], which have a low affinity for corticosterone, are likely to be the main target for negative feedback [60]. We, therefore, examined the expression of GR in the hippocampus using immunohistochemistry and western blotting in control mice. GR protein was shown to be more abundant in the perisomatic CA1 region of dorsal hippocampus than ventral hippocampus (Fig. 4A–C). Consistent with this result, GR protein expression in dorsal CA1 region was significantly higher than in ventral CA1 region (Fig. 4D). It has been reported that a bath application of 100 nM corticosterone for 20 min is sufficient to occupy GRs in hippocampal CA1 neurons, resulting in increased Ca^2+^ current amplitude, which was prevented by pretreatment with the GR antagonist [61, 62]. We previously reported that in vitro or in vivo block of the sarcoplasmic/endoplasmic reticulum Ca^2+^-ATPase (SERCA) pumps in CA1 neurons increases perisomatic *I*_h_ [43, 44, 63]. Given that dorsal CA1 region had more GR expression than ventral CA1 region, we investigated if corticosterone had distinct physiological effects on CA1 neurons in dorsal and ventral hippocampi. Corticosterone had physiological effects (e.g., changes in R_in_ and the number of action potentials) after 20 min, and the effects were reversible over time (Data not shown). We, therefore, treated corticosterone for 20 min in all our experiments. In dorsal CA1 neurons, corticosterone had no effect on RMP (Fig. 4E, F), but lowered R_in_ at RMP (Fig. 4E, G) and at −65 mV (Fig. S5A, B) compared with baseline. Furthermore, the number of action potentials elicited by depolarizing current steps at RMP (Fig. 4K, L) and at −65 mV (Fig. S5E, F) was significantly decreased compared with baseline. However, there were no significant differences in RMP (Fig. 4H, I), R_in_ at RMP (Fig. 4H, J) and at −65 mV (Fig. S5C, D), and the number of action potentials at RMP (Fig. 4M, N) and at −65 mV (Fig. S5G, H) in ventral CA1 neurons before and after treatment with 100 nM corticosterone. Although corticosterone at a concentration of 100 nM had different physiological effects on hippocampal CA1 neurons, we wondered if other corticosterone concentrations did as well. We then examined the same electrophysiological parameters (i.e., R_in_, and APs) with different concentrations of corticosterone (i.e., 10 nM and 1 μM). We found that neither 10 nM corticosterone (Fig. S6A, J) nor 1 μM corticosterone (Fig. S7A, J) had effects on dorsal or ventral CA1 neurons (Fig. 4O–Q). We, therefore, used 100 nM corticosterone for all our experiments.

### Corticosterone increased *I*_h_ as well as TRIP8b and HCN1 protein expression

**Fig. 5:**
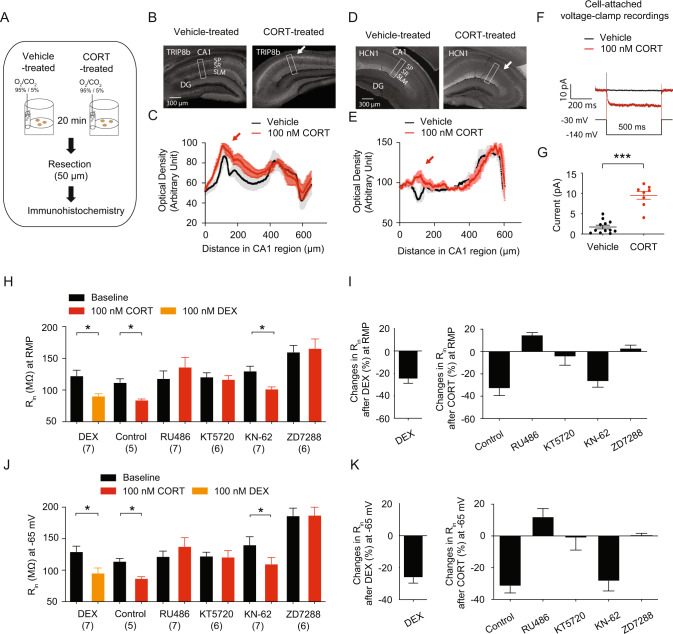
The GR, HCN channels, and the PKA pathway were all involved in corticosterone-induced upregulation of functional *I*_h_. **A** Illustration depicting the acute corticosterone treatment in the dorsal hippocampus. **B** Representative dorsal hippocampal slices immunolabeled with antibody against TRIP8b. Rectangle boxes depict the region of the slice used for quantification of the optical density. The arrows indicate increased perisomatic TRIP8b protein expression. **C** Quantification of TRIP8b protein expression from the perisomatic region to the distal dendritic region of CA1 from the dorsal hippocampus. **D** Representative dorsal hippocampal slices immunolabeled with antibody against HCN1. Rectangle boxes depict the region of the slice used for quantification of the optical density. The arrows indicate increased perisomatic HCN1 protein expression. **E** Quantification of HCN1 protein expression from the perisomatic region to the distal dendritic region of CA1 from the dorsal hippocampus. **F**, **G** We performed cell-attached voltage-clamp recordings. **F** Representative maximal *h* current traces in response to a 500-ms hyperpolarizing voltage step (−140 mV). **G** *I*_h_ was significantly elevated in the dorsal CA1 neurons from corticosterone treatment compared with those from the vehicle-treated group. Dexamethasone reduced R_in_ at RMP (**H**) and at −65 mV (**J**) in dorsal CA1 neurons. Corticosterone-induced decrease in R_in_ at RMP (**H**) and at −65 mV (**J**) was blocked by RU 486, KT5720, and ZD7288. Changes in R_in_ at RMP (**I**) and at −65 mV (**K**) were blocked by RU 486, KT5720, and ZD7288. Data are expressed as mean ± SEM.

At RMP, HCN channels are active, leading to a depolarized membrane potential and a decrease in R_in_ [38]. Furthermore, we observed the effects of corticosterone on dorsal CA1 neurons/region within 30 min, indicating that gene expression was not a factor. TRIP8b (also known as PEX5R) regulates HCN channel function and trafficking in the brain [64–66]. We investigated whether corticosterone-induced increased functional *I*_h_ (i.e., decreased R_in_) in dorsal CA1 neurons was due to the increased HCN channel trafficking. We used a 50 μm thick resection from either vehicle- or corticosterone-pretreated dorsal hippocampal slices (300 μm thick) for immunohistochemistry (Fig. 5A). Indeed, TRIP8b was significantly increased in the perisomatic CA1 region of dorsal hippocampus following corticosterone pretreatment (Fig. 5B, C). Consistent with this result, HCN1 protein expression was significantly increased in the perisomatic CA1 region of dorsal hippocampus (Fig. 5D, E). Furthermore, cell-attached recordings revealed that *I*_h_ was significantly increased in dorsal CA1 neurons (Fig. 5F, G), which was consistent with corticosterone-induced elevation of TRIP8b and HCN1 protein expression in the perisomatic CA1 region of dorsal hippocampus.

### Corticosterone-induced upregulation of functional *I*_h_ was mediated by the GR, HCN channels, and the PKA but not the CamKII pathway

We next investigated whether the effects of corticosterone on functional *I*_h_ were via the GR activation. Dexamethasone is a selective GR agonist (K_d_ = 4.6 nM) [67, 68]. We examined if dexamethasone mimicked the effects of corticosterone. We employed 100 nM dexamethasone based on physiological responses in hippocampal CA1 neurons [69]. We tested if the lower concentration (25 nM) of dexamethasone had physiological effects on CA1 neurons before using the 100 nM dexamethasone. RMP (Fig. S8A, B), R_in_ at RMP (Fig. S8A, C), and action potential firing at RMP (Fig. S8D, E) were unaffected by 25 nM dexamethasone, but 100 nM dexamethasone reduced R_in_ at RMP (Figs. 5H, I and S8F, H), R_in_ at −65 mV (Fig. 5J, K), and action potential firing at RMP (Fig. S8I, J) without affecting RMP (Fig. S8F, G). RU 486 (IC50 = 2.6 nM) is a potent selective antagonist of GR receptors [70]. 500 nM RU 486 is sufficient to block corticosterone-mediated physiological effects on hippocampal CA1 neurons [62]. We used RU 486 at a concentration of 500 nM. As a control experiment, 100 nM corticosterone lowered R_in_ at RMP (Fig. 5H, I), and at −65 mV (Fig. 5J, K). When dorsal CA1 neurons were pretreated with 500 nM RU 486, corticosterone had no effects on R_in_ at RMP (Figs. 5H, I and S9A–C), and at −65 mV (Fig. 5J, K). Furthermore, corticosterone-induced decreased neuronal excitability at RMP was blocked by GR antagonist RU 486 (Fig. S9D, E). These results indicate that corticosterone-induced changes in functional *I*_h_ were mediated by the activation of GR. The PKA pathway is involved in non-genomic glucocorticoid-GR effects [71, 72]. In addition, *I*_h_ maximal conductance is modulated by PKA-dependent phosphorylation [73]. We, therefore, investigated if the PKA pathway was required for the rapid corticosterone-induced changes in functional *I*_h_. KT5720 (Ki = 60 nM) is a potent, selective inhibitor of PKA signaling [74]. As previously reported, we employed KT5720 at a concentration of 500 nM [63, 75]. In the presence of KT5720, we investigated the effects of corticosterone on functional *I*_h_. R_in_ at RMP (Figs. 5H, I and S10A–C), R_in_ at −65 mV (Fig. 5J, K), and the number of action potentials at RMP (Fig. S10D, E) were unaffected following corticosterone treatment in dorsal CA1 neurons, suggesting that corticosterone-mediated effects on functional *I*_h_ were dependent on the PKA pathway. Given that 100 nM corticosterone enhanced calcium current amplitude via the GR activation [61], we next investigated whether the CamKII was required for the effects on functional *I*_h_ in dorsal CA1 neurons. KN-62 is a selective inhibitor of CamKII with ki of 0.9 μM [76]. 5 μM KN-62 blocks the CamKII pathway, as previously described [63, 77]. We, therefore, used KN-62 at a concentration of 5 μM. Despite KN-62 blocking the CamKII signaling pathway, we observed changes in R_in_ at RMP (Fig. 5H, I and S11A–C), R_in_ at −65 mV (Fig. 5J, K), and the number of action potentials at RMP (Fig. S11D, E) following corticosterone treatment in dorsal CA1 neurons. These results imply that corticosterone-mediated changes in functional *I*_h_ was not mediated by the CamKII pathway. We next examined if corticosterone-induced changes in functional *I*_h_ were mediated directly by HCN channels. ZD7288 is a potent HCN channel blocker at a concentration range of 10 to 20 μM [34, 44]. The effects of corticosterone in dorsal CA1 neurons were determined in the presence of the ZD7288. In a previous study, we observed that using ZD7288 in the bath caused nonspecific effects, such as progressive depolarization 10–15 min after the hyperpolarization of V_m_ [34]. To circumvent this, we used ZD7288 in the pipette (20 μM) instead of the bath application. As predicted from a blockage of HCN channels, ZD7288 caused a significant hyperpolarization of the RMP (Fig. S12A), increased R_in_ at RMP (Fig. S12B) and at −65 mV (Fig. S12C) compared to control group. When HCN channels were blocked, the corticosterone had no effects on RMP (Fig. S12D, E), R_in_ at RMP (Figs. 5H, I and S12D, F), R_in_ at −65 mV (Fig. 5J, K), and the number of action potentials at −65 mV (Fig. S12G, H).

### Susceptible mice displayed a persistent social avoidance, which was linked to increased *I*_h_ and were insensitive to corticosterone effects

**Fig. 6:**
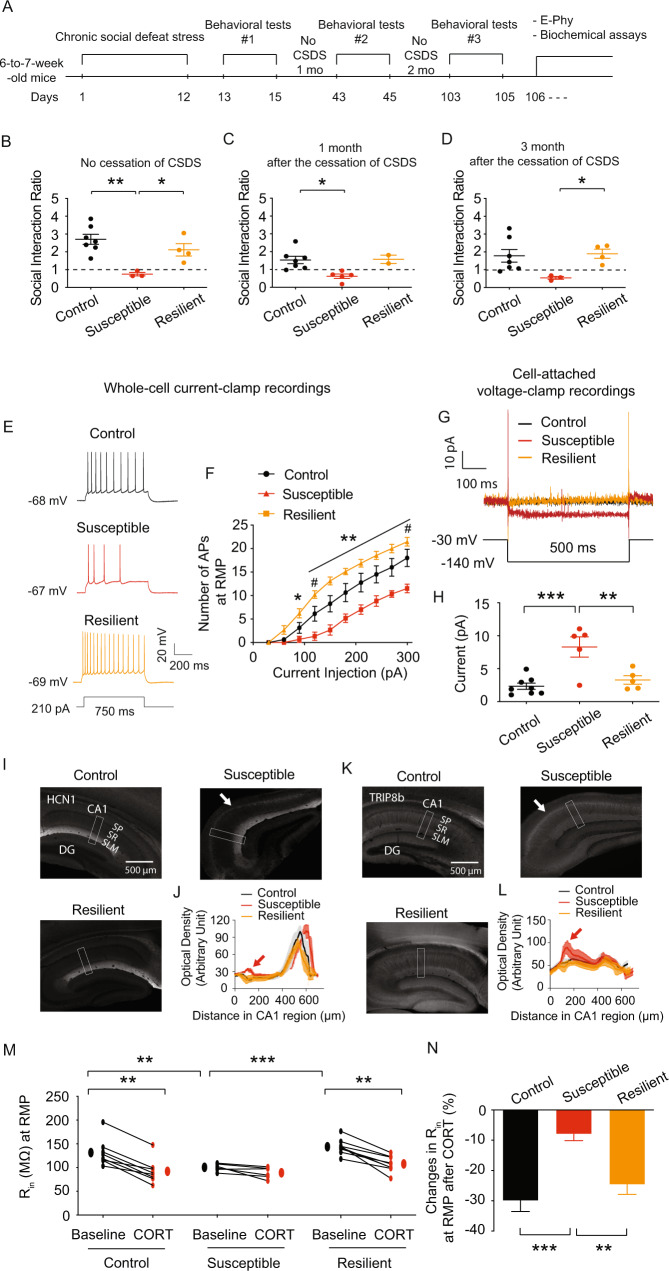
Susceptible mice re-exposed to novel aggressor mice displayed a persistent social avoidance, which was associated with elevated *I*_h_ and was insensitive to corticosterone effects. **A** Timeline of CSDS, behavioral tests, electrophysiology, and biochemical assay. **B** CSDS produced the susceptible and resilient phenotype during the social interaction test. After 1 month (**C**) or 3 months (**D**) of no CSDS, susceptible mice showed persistent social avoidance during the social interaction test. **E**, **F** We performed whole-cell current-clamp recordings. **E** Representative voltage responses with depolarizing current step (210 pA; 750 ms) at RMP in dorsal CA1 neurons. **F** Dorsal CA1 neurons of susceptible group had lower action potential firing than control group, whereas the resilient group had higher action potential firing. **G**, **H** We performed cell-attached voltage-clamp recordings. **G** Representative maximal *h* current traces in response to a 500-ms hyperpolarizing voltage step (−140 mV). **H** *I*_h_ was significantly elevated in the dorsal CA1 neurons from susceptible group compared with those from the control and resilient mice. Representative dorsal hippocampal slices immunolabeled with antibody against HCN1 (**I**) and TRIP8b (**K**). Rectangle boxes depict the region of the slice used for quantification of the optical density. The arrows indicate increased perisomatic HCN1 and TRIP8b protein expression. Quantification of HCN1 (**J**) and TRIP8b (**L**) protein expression from the perisomatic region to the distal dendritic region of CA1 from the dorsal hippocampus. **M**, **N** We performed whole-cell current-clamp recordings. **M** Corticosterone reduced R_in_ at RMP of the dorsal CA1 neurons in the control and resilient groups, but had no effect on R_in_ in susceptible group. **N** Changes in R_in_ at RMP were much lower in susceptible group compared to the control and resilient groups. Data are expressed as mean ± SEM.

One of the most distressing symptoms of PTSD is the recurrence of the experience [1]. A longitudinal study of PTSD patients reveals that they have PTSD symptoms even 50 or more years after their traumatic experience [78, 79]. In CSDS paradigm, naive mice were repeatedly exposed to aggressor mice. After 12 days in this physically and emotionally stressful environment, naive mice showed susceptible (i.e., increased social avoidance) and resilient (i.e., normal-like social interaction) phenotypes during the social interaction test. These susceptible mice had a painful recollection of aggressor mice. Furthermore, these impaired behavioral results were linked to (1) decreased neuronal excitability, (2) increased perisomatic HCN1 protein expression, and (3) elevated *I*_h_ in dorsal but not ventral CA1 region/neurons. We, first, examined if susceptible mice exposed to novel aggressor mice re-experienced the trauma during the social interaction test. Because the difference in human age between mice aged 3 months and 6 months is over 10 years [80], we chose re-exposure periods of 1 month and 3 months with singly housed 3-month-old CSDS-treated and control mice. As a result, we can see whether (1) CSDS-induced social avoidance lasted for a long period and (2) social avoidance was linked to pathological changes in *I*_h_. CSDS was subjected to male mice aged 6 to 7 weeks for 12 days (Fig. 6A). During the social interaction test, CSDS produced susceptible and resilient phenotypes (Figs. 6B and S13A). Susceptible mice demonstrated re-experiencing trauma such as social avoidance after one month (Figs. 6C and S13A) and three months (Figs. 6D and S13A) without CSDS. Because dorsal CA1 neurons from susceptible mice had lower neuronal excitability than the control and resilient mice, we investigated the physiological effects on dorsal CA1 neurons following behavioral tests after 3 months without CSDS. Acute dorsal hippocampal slices were prepared after behavioral tests [43]. After acute hippocampal slicing, we set aside tissue samples for immunohistochemistry. Electrophysiological data and protein expression might therefore be connected. Dorsal CA1 neurons from susceptible group displayed lower neuronal excitability than the control and resilient groups (Fig. 6E, F), which was consistent with earlier findings (Fig. 2D, E). Interestingly, resilient group showed higher neuronal excitability of dorsal CA1 neurons than that of the control and susceptible groups (Fig. 6E, F). In comparison to the control and resilient groups, cell-attached patch-clamp recordings indicated a substantial increase in *I*_h_ in dorsal CA1 neurons from susceptible group (Fig. 6G, H). We next investigated whether HCN1 and TRIP8b protein expression were changed along the somatodendritic axis of dorsal CA1 neurons. HCN1 (Fig. 6I, J) and TRIP8b (Fig. 6K, L) protein expression in susceptible group were considerably increased in the perisomatic CA1 region of the dorsal hippocampus, consistent with increased *I*_h_. Corticosterone increased TRIP8b and HCN1 protein expression in vitro hippocampus slices, leading in an increase in *I*_h_ through GRs and HCN channels (Fig. 5). We next investigated whether corticosterone-induced changes in functional *I*_h_ (i.e., R_in_) were altered between groups. RMP was unaffected by corticosterone in any of the groups (Fig. S14A, B). Before treating corticosterone, dorsal CA1 neurons from susceptible group had lower R_in_ at RMP (Fig. 6M) and at −65 mV (Fig. S14C) than the control and resilient group, consistent with elevated *I*_h_. When dorsal CA1 neurons were treated with corticosterone, control and resilient groups displayed reduced R_in_ at RMP (Fig. 6M, N) and at −65 mV (Fig. S14C, D). Surprisingly, corticosterone had no additional effect on R_in_ at RMP (Fig. 6M, N) and at −65 mV (Fig. S14C, D) in dorsal CA1 neurons from susceptible group.

## Discussion

We found that social avoidance and impaired spatial working memory were seen in susceptible mice, which were linked to increased perisomatic HCN1 protein expression, higher *I*_h_, and lower neuronal excitability in the dorsal but not ventral CA1 neurons. In control mice, bath application of corticosterone had effects similar to those in susceptible mice. Corticosterone in the bath reduced R_in_, lowered neuronal excitability, increased TRIP8b and HCN1 protein expression, and elevated *I*_h_ in the dorsal but not ventral CA1 region/neurons. These corticosterone-induced alterations in functional *I*_h_ depended on the GR, HCN channels, and the PKA but not the CamKII pathway. Finally, susceptible mice re-exposed to new aggressor mice after one month or three months without CSDS displayed social avoidance. This persistent behavioral deficit in susceptible mice was associated with increased perisomatic TRIP8b and HCN1 protein expression, elevated *I*_h_, reduced R_in_, and lower neuronal excitability in the dorsal CA1 region/neurons. Furthermore, alterations in functional *I*_h_ caused by corticosterone-GR activation were not observed in the dorsal CA1 neurons of susceptible mice but were seen in control and resilient mice.

PTSD is a severe mental condition that can develop due to life-threatening events. One of the most common PTSD symptoms is avoidance. Experiential avoidance is the key to making a traumatized person more likely to develop and maintain PTSD [11]. CSDS is a rodent model of psychosocial stress that causes social avoidance and anhedonia [14], indicators of human PTSD. Interestingly, some repeatedly socially defeated mice have susceptible phenotypes, while others do not [14], similar to human PTSD. Although women are twice as likely as men to suffer from PTSD [81], using the CSDS paradigm with female mice/rats is complicated and unreliable [82]. We attempted to use female mice in CSDS. However, ovariectomized female CD-1 mice had a considerably lower frequency of physical attack, a shorter attack duration, and a longer attack latency than male aggressor CD-1 mice (Data not shown). Unlike the female mice in CSDS, chronic socially defeated male mice exhibited anxiogenic-like behavior in the elevated plus-maze test. During the social interaction test, some defeated male mice had a traumatic memory of aggressor male mice, which caused them to avoid social engagement (i.e., social avoidance). The social avoidance persisted for at least three months when susceptible male mice were reintroduced to new aggressor male mice. Furthermore, in the Y maze test, susceptible male mice had worse spatial working memory. Overall, our animal model produced behavioral deficits in some populations of traumatic event-exposed male mice, comparable to those seen in human PTSD.

We initially examined the neuronal excitability of dorsal and ventral CA1 neurons in control, susceptible, and resilient male mice since hippocampal hypoactivity has been linked to human PTSD [83]. After 12 days of CSDS, susceptible mice had decreased R_in_ and lower neuronal excitability in the dorsal but not ventral CA1 neurons compared to control and resilient groups. In addition, dorsal CA1 neurons of the susceptible group showed increased HCN1 protein expression and elevated *I*_h_. HCN channels are partially active at the resting membrane potential, producing a non-inactivating, depolarizing inward current. Given that its reversal potential is around −30 mV, *I*_h_ should exert an excitatory action by depolarizing the membrane potential toward the action potential threshold. However, HCN1 channels are highly enriched in distal dendrites of the hippocampal CA1 region, providing a paradoxical inhibitory action such as reduction of synaptic integration and decreased neuronal excitability by input resistance [38]. This paradoxical inhibitory effect of *I*_h_ might depend on the interaction with other ionic conductances (e.g., delayed-rectifier M-type K^+^ channels) in hippocampal and cortical pyramidal neurons [84]. Surprisingly, after three months without CSDS, dorsal CA1 neurons from susceptible mice had lower R_in_ and neuronal excitability. However dorsal CA1 neurons from resilient mice had higher R_in_ and neuronal excitability than control and susceptible groups (Fig. 6). Compared to the results from Figs. 1 and 2 (i.e., no cessation of CSDS), hypo-neuronal excitability of dorsal CA1 neurons was persistent in susceptible mice. In contrast, resilient mice had hyper-neuronal excitability of dorsal CA1 neurons. Further research is needed to understand how mice exposed to traumatic events develop resilient phenotypes associated with increased R_in_ and neuronal excitability. Regardless of this, neuronal excitability of dorsal CA1 neurons might be the cellular mechanism underpinning the development of susceptibility and resilience to social avoidance. Consistent with our findings, mice infused with corticosterone in the dorsal CA1 region produce PTSD-like memories such as emotional hypermnesia and contextual amnesia [85–87]. Normal mice display PTSD-like memory when dorsal CA1 neurons are inhibited by optogenetic inhibition during stress. On the other hand, optogenetic activation of dorsal CA1 neurons during stress prevents PTSD-like memory [85]. It has been reported that the dorsal hippocampus has more GR protein expression than the ventral hippocampus [88, 89]. Consistent with these reports, we also found that GR protein expression was more significant in the dorsal hippocampus than in the ventral hippocampus. Interestingly, GR protein was highly concentrated in the dorsal CA1 perisomatic area but not in the ventral CA1 region (Fig. 4). In our previous studies, blocking the SERCA pumps in dorsal CA1 neurons, which elevate intracellular calcium levels, increases *I*_h_ and functional *I*_h_ (i.e., reduced R_in_ and lower neuronal excitability) [43, 63]. Corticosterone-GR activation has been shown to enhance Ca^2+^ current amplitude in rat/mouse hippocampal CA1 neurons [61, 62]. Indeed, dorsal CA1 neurons had decreased R_in_ and lower neuronal excitability following corticosterone treatment (Fig. 4). Dexamethasone, a selective GR agonist, elicited physiological responses in dorsal CA1 neurons similar to those of corticosterone. When dorsal CA1 neurons were pretreated with GR antagonist (RU 486), the effects of corticosterone on R_in_ and neuronal excitability were blocked (Fig. 5). Furthermore, decreased R_in_ and neuronal excitability caused by corticosterone treatment were blocked by the HCN channel blocker (ZD7288) (Fig. 5). These findings indicate that corticosterone reduced R_in_ and neuronal excitability through activating GR and HCN channels. HCN1 protein is abundant in the CA1 area of the hippocampus, with a gradient of increasing channel density from the soma to the distal apical dendrites [38, 39]. The aberrant HCN1 protein subcellular distribution along the somatodendritic axis of CA1 neurons is linked to pathogenic events in the animal models of depression and temporal lobe epilepsy [43, 44, 90]. Because corticosterone reduced R_in_ and neuronal excitability of dorsal CA1 neurons from control mice, we looked to see if corticosterone affected the protein expression of HCN1 and TRIP8b, the primary auxiliary subunit of HCN channels. Pretreatment with corticosterone for 20–30 min increased perisomatic TRIP8b protein expression, which led to higher perisomatic HCN1 protein expression. Cell-attached recordings and whole-cell voltage-clamp recordings showed that corticosterone increased *I*_h_ in the dorsal CA1 neurons. PKA-dependent phosphorylation is involved in the control of *I*_h_ maximal conductance [73]. When dorsal CA1 neurons were pretreated with the PKA inhibitor (KT5720), the effects of corticosterone on functional *I*_h_ were blocked (Fig. 5). As a result, rapid effects of corticosterone on functional *I*_h_ were mediated by the expression of TRIP8b and HCN1 protein and the PKA pathway. When dorsal CA1 neurons from control mice were treated with corticosterone, R_in_ and neuronal excitability were both decreased, which was similar to the results from susceptible mice. Furthermore, dorsal CA1 neurons from the susceptible mice had no further changes in functional *I*_h_ (i.e., R_in_) following corticosterone treatment, whereas control and resilient mice did. These findings imply that elevated *I*_h_ in the dorsal CA1 neurons from susceptible mice occluded corticosterone-induced upregulation of functional *I*_h_.

Selective serotonin reuptake inhibitors are Food and Drug Administration-approved medications for treating PTSD that primarily act by targeting the monoamine system and raising serotonin levels in limbic brain regions, such as the hippocampus. However, after the initial pharmacologic treatment, only around 20–30% of patients with PTSD satisfy remission criteria [91, 92]. Despite its significant side effects, such as psychotomimetic symptoms and the danger of drug abuse, (R,S)-ketamine has an immediate therapeutic impact on human PTSD [93, 94]. (S)-ketamine is an HCN1 inhibitor [44, 95] and an antagonist of the N-methyl-D-aspartate receptor [96]. Pretreatment with (S)-ketamine before the beginning of CUS prevented aberrant behaviors (i.e., anxiogenic- and depressive-like behaviors) and neuropathological alterations caused by CUS (i.e., upregulation of perisomatic *I*_h_ and a decrease in neuronal excitability) [43, 44]. A reduction of HCN1 protein expression in the dorsal CA1 region (about 30% of the dorsal CA1 region) before the CUS-induced onset of depression and anxiety is sufficient to provide resilient effects to CUS [43]. Corticosterone-GR activation-induced lower neuronal excitability was blocked by the HCN channel blocker (Fig. 6). This finding might explain why knockdown of HCN1 protein expression or pretreatment of (S)-ketamine before the onset of depression and anxiety provides resilient effects [43, 44].

In conclusion, we have demonstrated that susceptible mice displayed social avoidance and impaired spatial working memory. In dorsal CA1 neurons, susceptible mice reduced neuronal excitability, whereas resilient mice had increased neuronal excitability. These results suggest that the neuronal excitability of dorsal CA1 neurons might be the cellular mechanism underlying the development of susceptibility and resilience to social avoidance. Acute corticosterone treatment in dorsal CA1 neurons from control mice, where perisomatic GR was strongly expressed, produced comparable effects, as seen by susceptible mice. These effects of corticosterone were mediated by the GR, HCN channels, and the PKA pathway. Importantly, corticosterone did not affect functional *I*_h_ in dorsal CA1 neurons from susceptible mice, while it affected the control and resilient groups. Our findings imply that abnormal subcellular expression of HCN1 protein along the somatodendritic axis of dorsal CA1 neurons contributes to a reduction in neuronal excitability and may be the molecular mechanism underpinning the development of susceptibility to social avoidance.

## Supplementary information

Supplementary Figures

Supplementary Table 1

Statistical Table
