# Supplementary material for: Glucocorticoid-glucocorticoid receptor-HCN1 channels reduce neuronal excitability in dorsal hippocampal CA1 neurons
Source: Mol Psychiatry. 2022 Jul 15;27(10):4035–49. doi: 10.1038/s41380-022-01682-9 (PMC9718682; doi:10.1038/s41380-022-01682-9)
Supplement: Supplementary file 1 — Supplementary Figures [file 41380_2022_1682_MOESM1_ESM.pdf]

Supplementary Figure S1

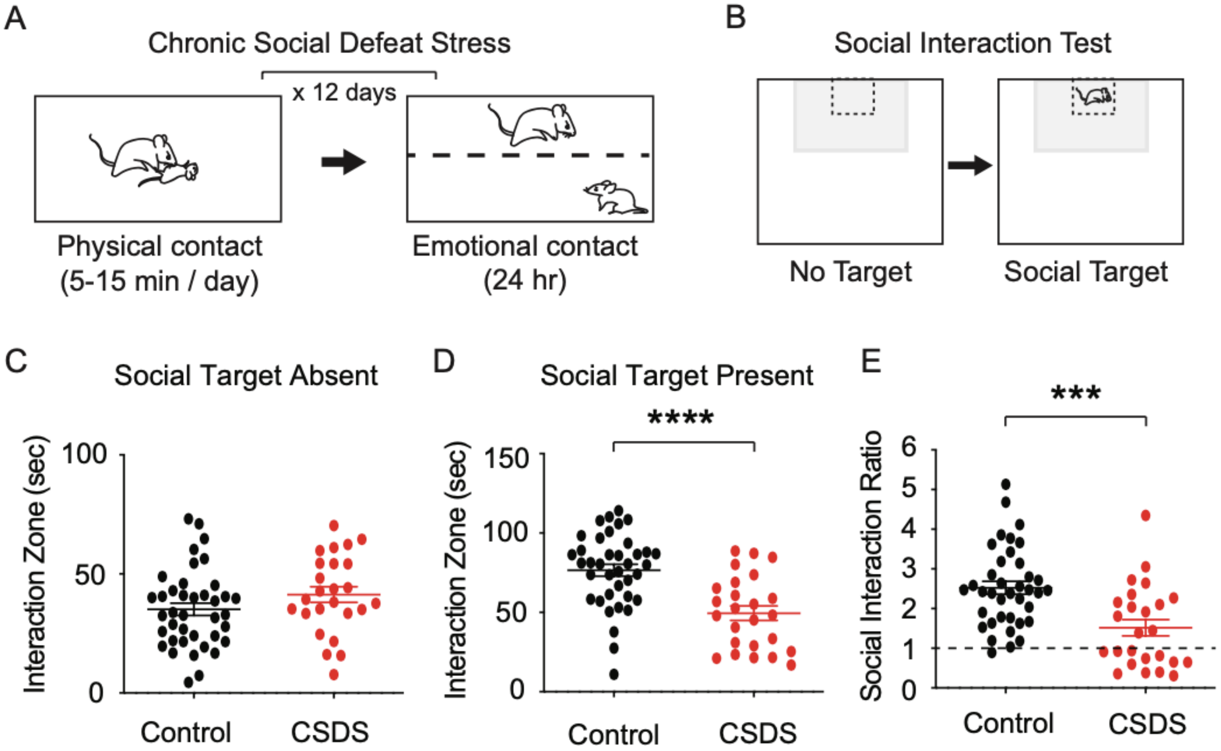

Supplementary Figure S1. The social interaction ratio was decreased in CSDS mice. (A) Illustration depicting the chronic social defeat stress. (B) Illustration depicting the social interaction test. (C) During the social interaction test, both control and CSDS mice spent the same amount of time in the interaction zone without a social target. (D) The susceptible group spent less time in the interaction zone in the presence of a social target than the control and resilient groups. (E) CSDS mice demonstrated a lower social interaction ratio than control mice during the social interaction test. Control (n = 39) and CSDS (n=25). Data are expressed as mean ± SEM

Supplementary Figure S2

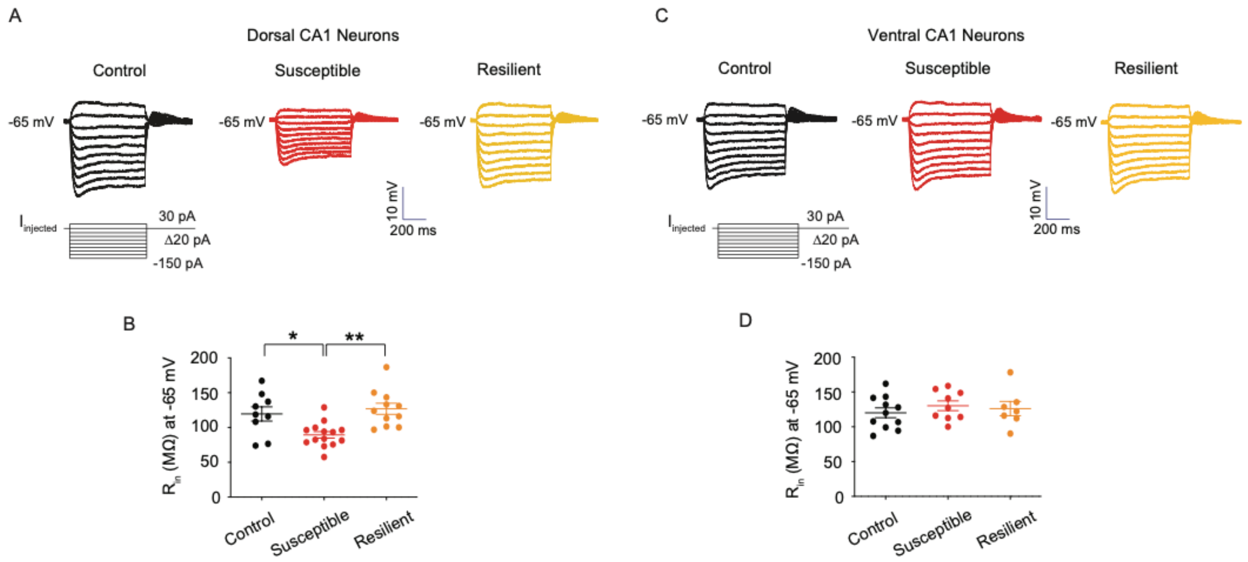

Supplementary Figure S2. The susceptible group reduced R_{in} at -65 mV in dorsal CA1 neurons but not in ventral CA1 neurons. (A and C) Representative voltage responses with step current commands ranging from -150 pA to +30 pA (Δ= 20 pA) at RMP. (B) The dorsal CA1 neurons of the susceptible group had lower R_{in} at -65 mV than the control and resilient groups. (D) There was no difference in R_{in} at -65 mV of ventral CA1 neurons between groups. Control (n=9), susceptible (n=14), and resilient (n=11). Data are expressed as mean ± SEM.

Supplementary Figure S3

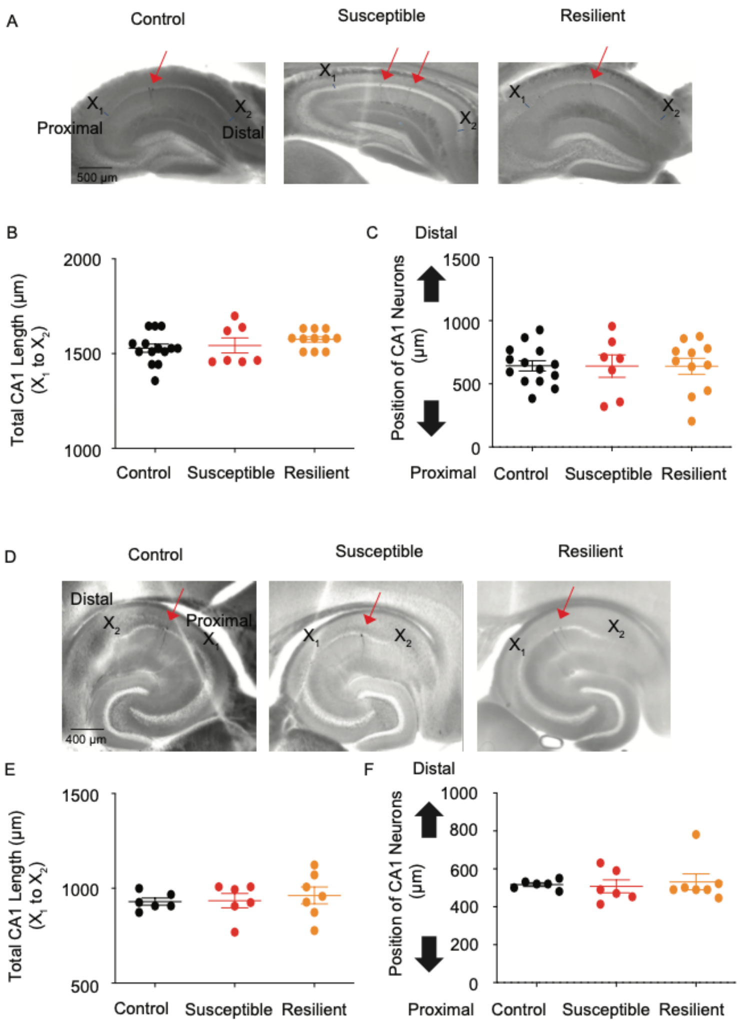

Supplementary Figure S3. The position of recorded CA1 neurons in dorsal and ventral hippocampus from control, susceptible, and resilient groups. (A and D) Representative dorsal (A) and ventral (D) slices in control, susceptible, and resilient groups. The arrows indicate the patched neurons in dorsal and ventral hippocampi. (B and E) The total length of CA1 was determined along the CA1 neuron layer between the CA2/CA1 border (X_1) and the CA1/subicular border (X_2). The position of recorded CA1 neurons along the CA1 region were determined by the distance (C and F). Dorsal : Control (n=14), susceptible (n=7), and resilient (n=11). Ventral : Control (n=6), susceptible (n=6), and resilient (n=7). Data are expressed as mean ± SEM.

Supplementary Figure S4

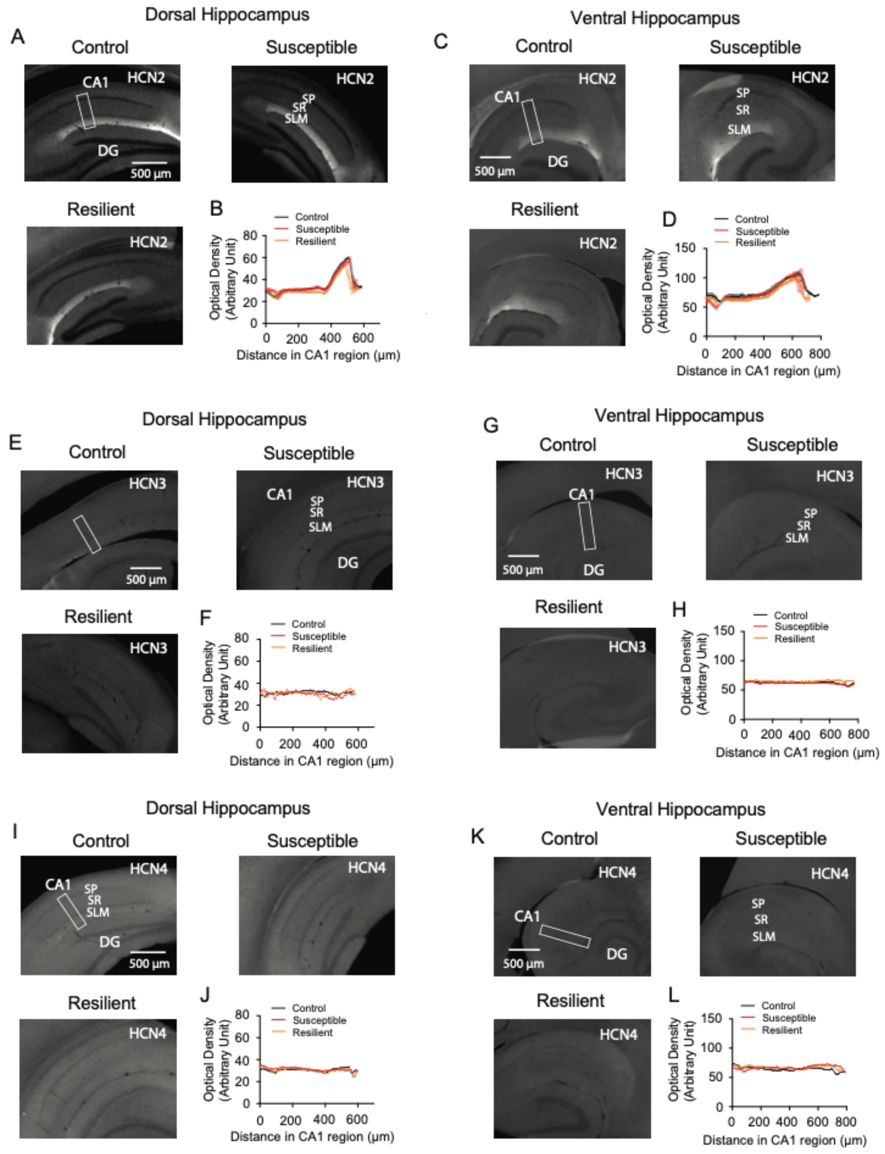

Supplementary Figure S4. HCN2, HCN3, and HCN4 protein expression in dorsal and ventral CA1 region from control, susceptible, and resilient groups. Representative dorsal (A, E, and I) and ventral (C, G, and K) hippocampal slices immunolabeled with antibody against HCN1, HCN2, and HCN3. Rectangle boxes depict the region of the slice used for quantification of the optical density. Quantification of HCN2, HCN3, and HCN4 protein expression from the perisomatic region to the distal dendritic region of CA1 from the dorsal (B, F, and J) and ventral (D, H, and L) hippocampus.

Supplementary Figure S5

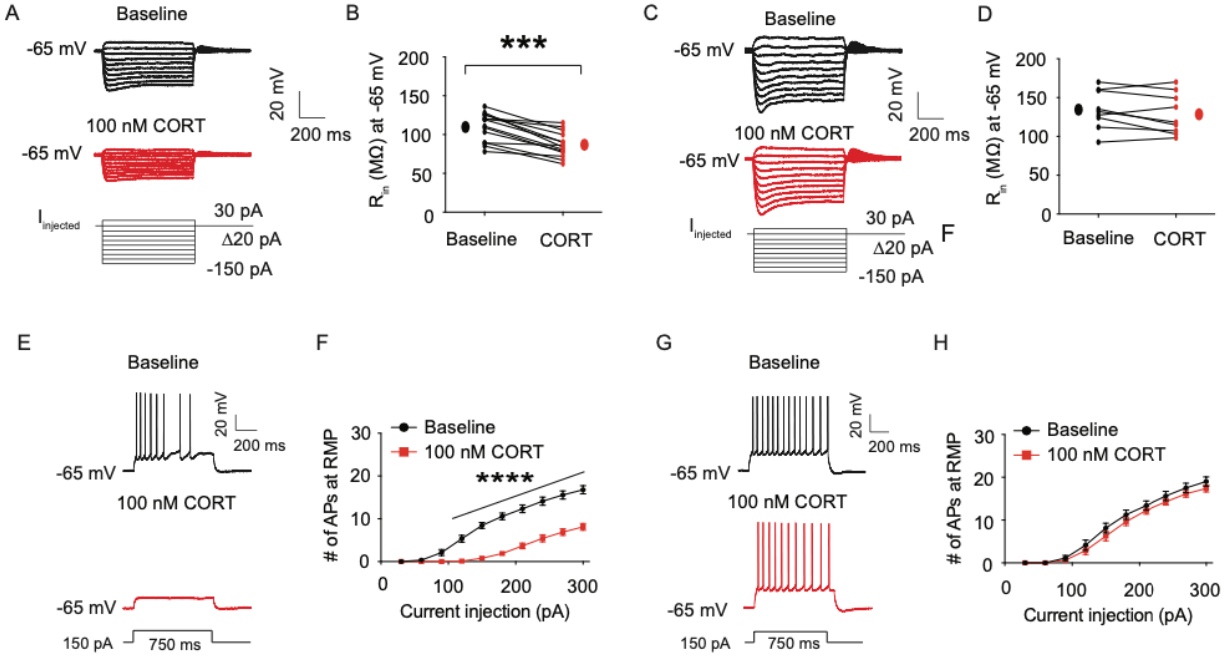

Supplementary Figure S5. 100 nM corticosterone reduced R_{in} at -65 mV and the number of action potentials at -65 mV in dorsal but not ventral CA1 neurons. (A and C) Representative voltage responses with step current commands ranging from -150 pA to +30 pA (Δ= 20 pA) at RMP before and after bath application of 100 nM corticosterone. (B and D) Corticosterone lowered R_{in} at -65 mV in dorsal (B) but not ventral (D) CA1 neurons. (E and G) Representative voltage responses with depolarizing current step (150 pA; 750 ms) at RMP in dorsal (E) and ventral (G) CA1 neurons. (E) Dorsal CA1 neurons had decreased action potential firing at -65 mV following corticosterone treatment. (H) Action potential firing at -65 mV was not altered in ventral CA1 neurons following corticosterone treatment. Dorsal (n=14) and ventral (n=9). Data are expressed as mean ± SEM.

Supplementary Figure S6

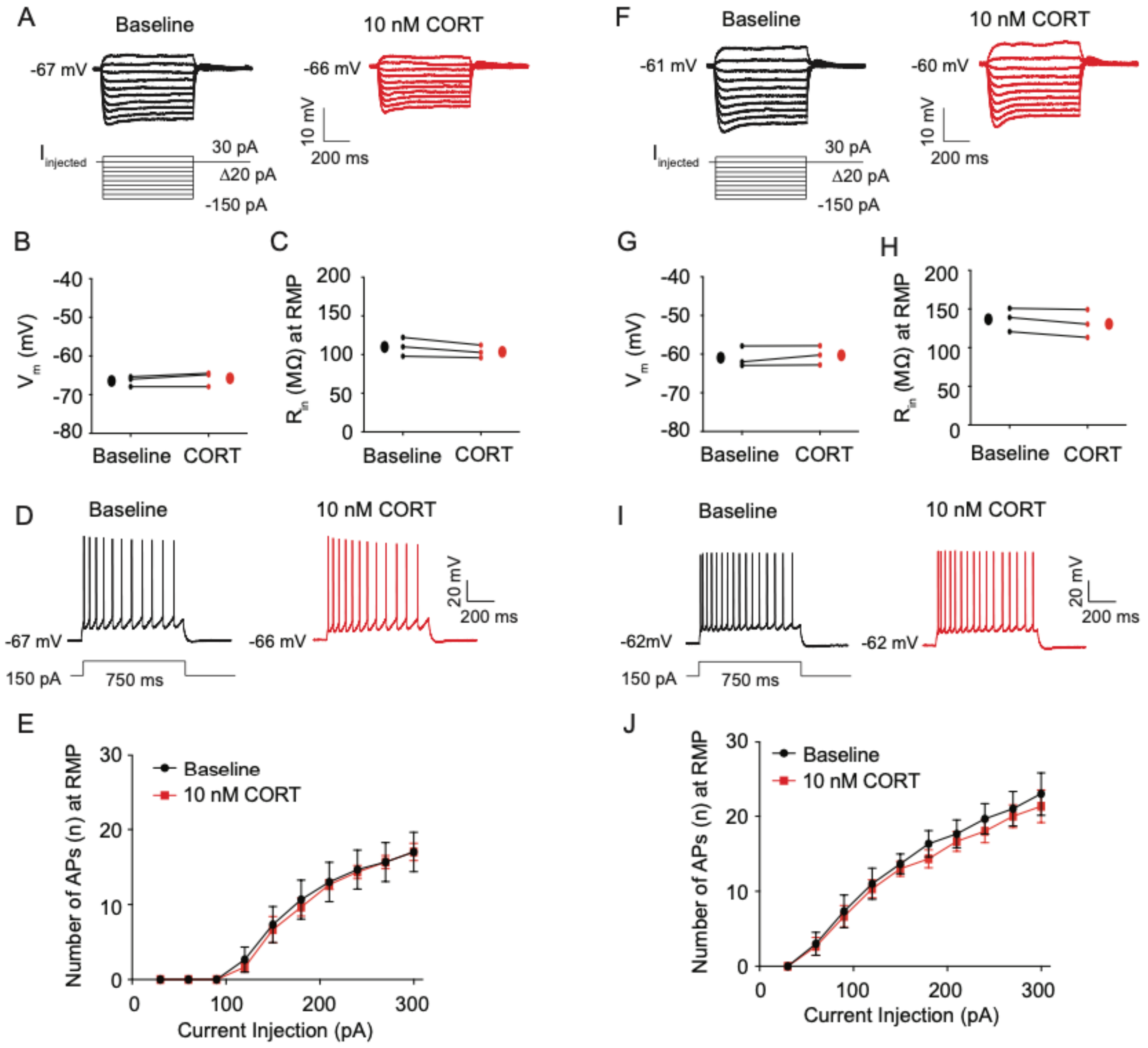

Supplementary Figure S6. 10 nM corticosterone had no effects on V_m, R_{in} at RMP, and the number of action potentials at RMP in dorsal and ventral CA1 neurons. (A and F) Representative voltage responses with step current commands ranging from -150 pA to +30 pA (Δ= 20 pA) at RMP before and after bath application of 10 nM corticosterone. Both dorsal and ventral CA1 neurons had no effects on V_m (B and G), and R_{in} at RMP (C and H). (D and I) Representative voltage responses with depolarizing current step (150 pA; 750 ms) at RMP before and after bath application of 10 nM corticosterone. Action potential firing was not altered following 10 nM corticosterone treatment in dorsal (E) and ventral (J) CA1 neurons. Dorsal (n=3) and ventral (n=3). Data are expressed as mean ± SEM.

Supplementary Figure S7

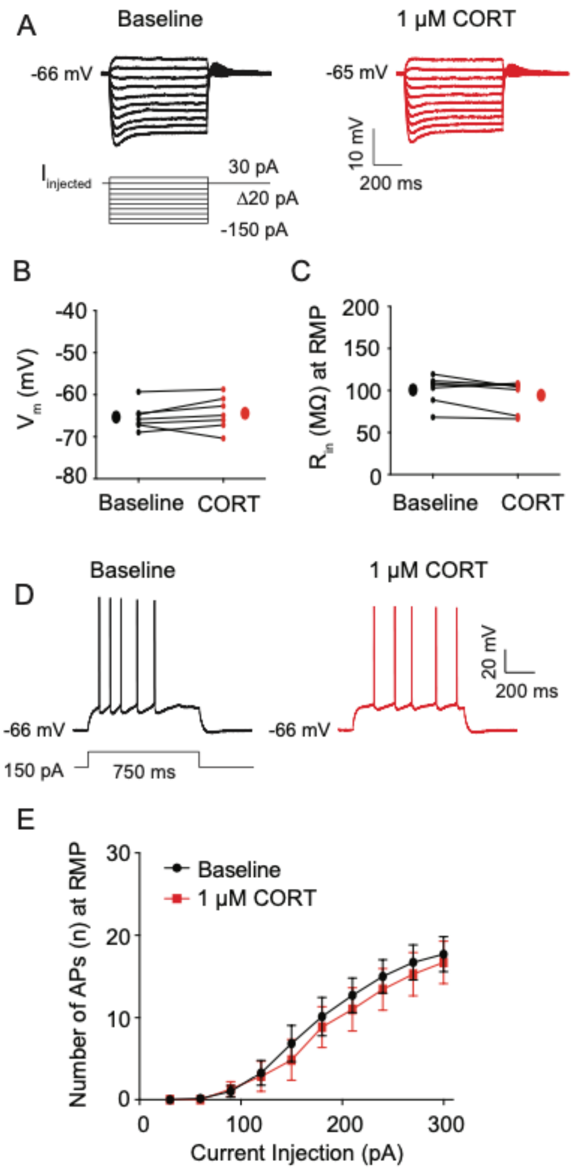

Supplementary Figure S7. 1 μM corticosterone had no effects on V_m, R_{in} at RMP, and the number of action potentials at RMP in dorsal and ventral CA1 neurons. (A and F) Representative voltage responses with step current commands ranging from -150 pA to +30 pA (Δ= 20 pA) at RMP before and after bath application of 1 μM corticosterone. Both dorsal and ventral CA1 neurons had no effects on V_m (B and G), and R_{in} at RMP (C and H). (D and I) Representative voltage responses with depolarizing current step (150 pA; 750 ms) at RMP before and after bath application of 1 μM corticosterone. Action potential firing was not altered following 1 μM corticosterone treatment in dorsal (E) and ventral (J) CA1 neurons. Dorsal (n=7) and ventral (n=6) CA1 neurons. Data are expressed as mean ± SEM.

Supplementary Figure S8.

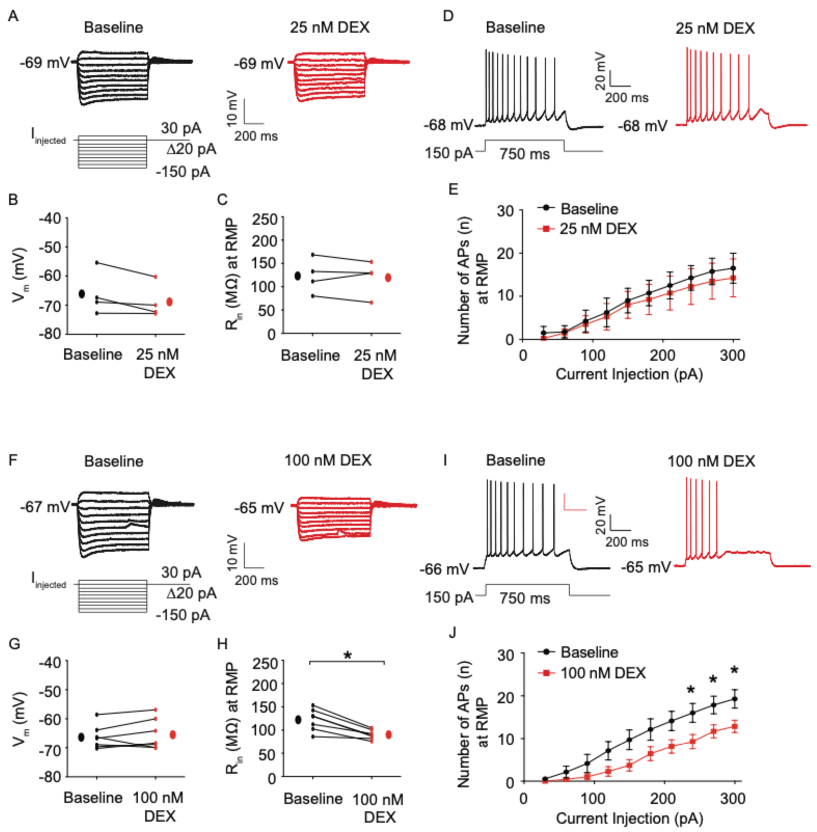

Supplementary Figure S8. 100 nM dexamethasone reduced R_{in} at RMP and lowered action potential firing at RMP. (A) Representative voltage responses with step current commands ranging from -150 pA to +30 pA (Δ= 20 pA) at RMP before and after bath application of 25 nM DEX. (B and C) 25 nM DEX had no effects on V_m (B) and R_{in} at RMP (C) in dorsal CA1 neurons. (D) Representative voltage responses with depolarizing current step (150 pA; 750 ms) at RMP before and after bath application of 25 nM DEX. Action potential firing was not altered following 25 nM DEX treatment in dorsal CA1 neurons. (F) Representative voltage responses with step current commands ranging from -150 pA to +30 pA (Δ= 20 pA) at RMP before and after bath application of 100 nM DEX. (G and H) 100 nM DEX had no effects on V_m (G), but lowered R_{in} at RMP (H) in dorsal CA1 neurons. (I) Representative voltage responses with depolarizing current step (150 pA; 750 ms) at RMP before and after bath application of 100 nM DEX. (J) 100 nM DEX reduced the number of action potentials in dorsal CA1 neurons. (n=7). Data are expressed as mean ± SEM.

Supplementary Figure S9

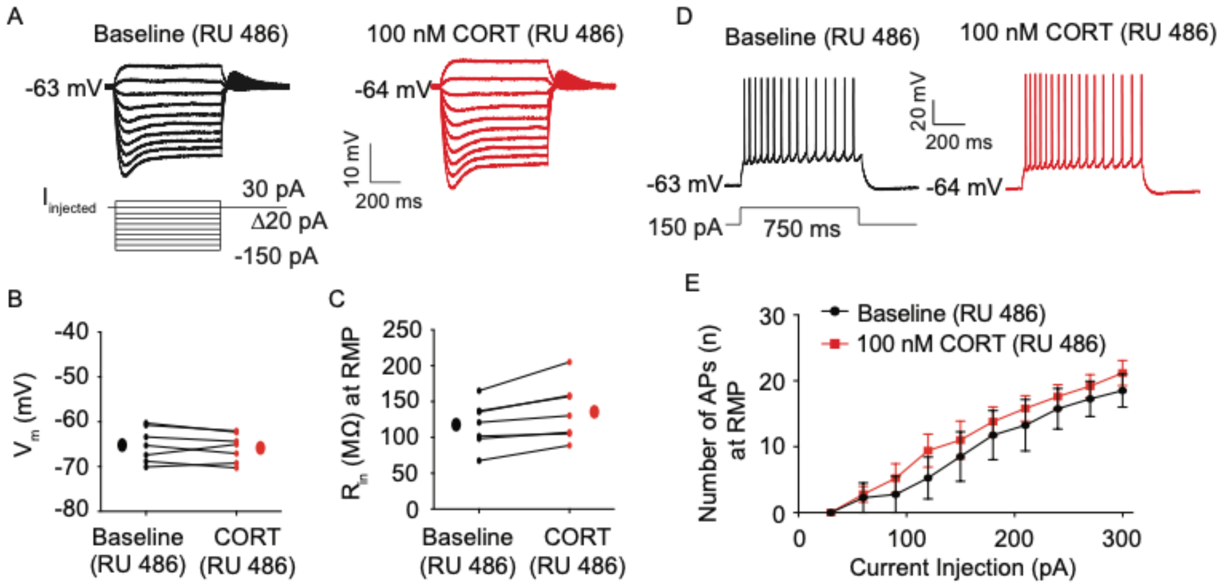

Supplementary Figure S9. Dorsal CA1 neurons had no effects on R_{in} at RMP and action potential firing following 100 nM corticosterone in the presence of GR antagonist RU 486. (A) Representative voltage responses with step current commands ranging from -150 pA to +30 pA (Δ= 20 pA) at RMP before and after bath application of 100 nM corticosterone in the presence of RU 486. (B and C) 100 nM corticosterone had no effects on V_m (B) and R_{in} at RMP (C) in dorsal CA1 neurons. (D) Representative voltage responses with depolarizing current step (150 pA; 750 ms) at RMP before and after bath application of 100 nM corticosterone in the presence of RU 486. (E) Action potential firing was not altered in dorsal CA1 neurons. (n=7). Data are expressed as mean ± SEM.

Supplementary Figure S10

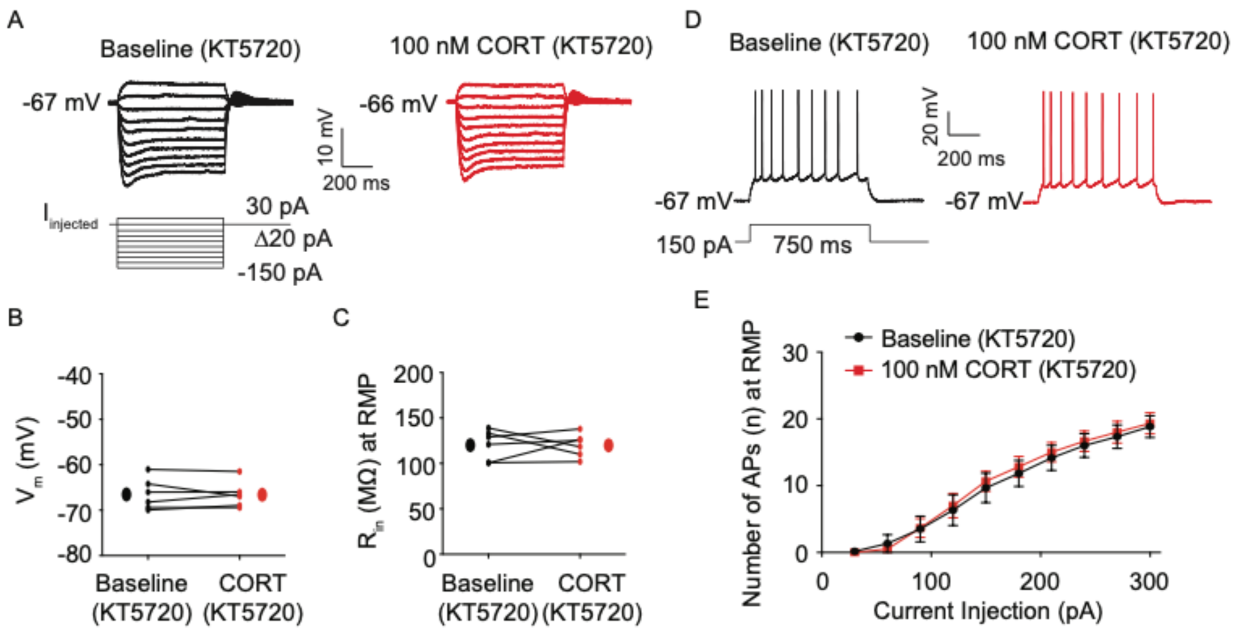

Supplementary Figure S10. Dorsal CA1 neurons had no effects on R_{in} at RMP and action potential firing following 100 nM corticosterone in the presence of PKA inhibitor (KT5720). (A) Representative voltage responses with step current commands ranging from -150 pA to +30 pA (Δ= 20 pA) at RMP before and after bath application of 100 nM corticosterone in the presence of KT5720. (B and C) 100 nM corticosterone had no effects on V_m (B) and R_{in} at RMP (C) in dorsal CA1 neurons. (D) Representative voltage responses with depolarizing current step (150 pA; 750 ms) at RMP before and after bath application of 100 nM corticosterone in the presence of KT5720. (E) Action potential firing was not altered in dorsal CA1 neurons. (n=6). Data are expressed as mean ± SEM.

Supplementary Figure S11

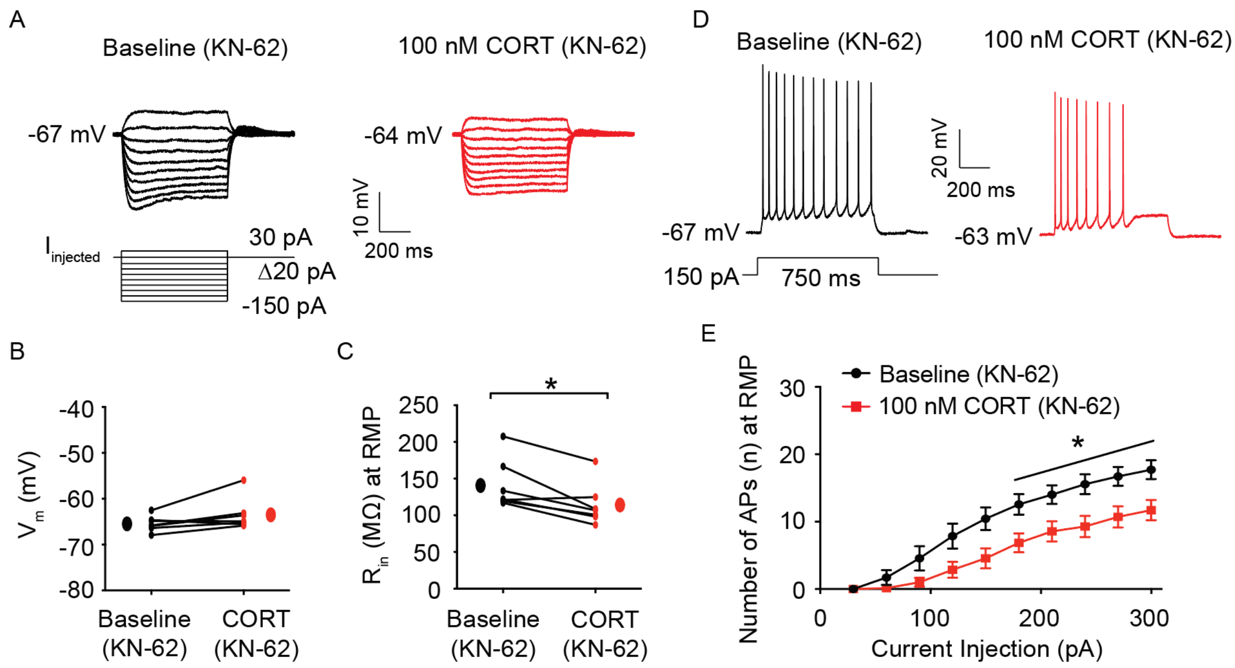

Supplementary Figure S11. Dorsal CA1 neurons had effects on R_{in} at RMP and action potential firing following 100 nM corticosterone in the presence of CamKII inhibitor (KN-62). (A) Representative voltage responses with step current commands ranging from -150 pA to +30 pA (Δ= 20 pA) at RMP before and after bath application of 100 nM corticosterone in the presence of KN-62. (B and C) 100 nM corticosterone had no effect on V_m (B), but reduced R_{in} at RMP (C) in dorsal CA1 neurons. (D) Representative voltage responses with depolarizing current step (150 pA; 750 ms) at RMP before and after bath application of 100 nM corticosterone in the presence of KN-62. (E) Action potential firing was reduced in dorsal CA1 neurons. (n=7). Data are expressed as mean ± SEM.

Supplementary Figure S12

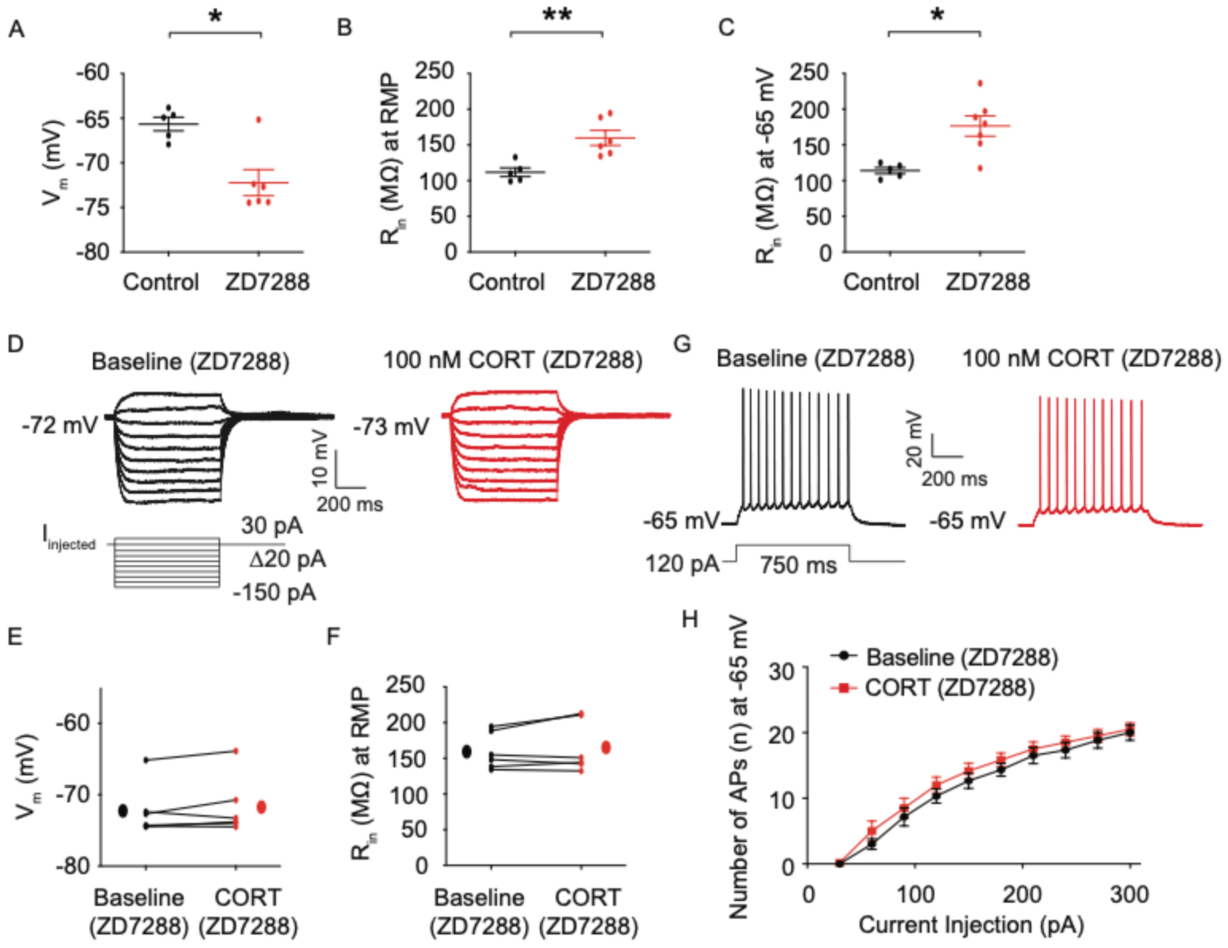

Supplementary Figure S12. Dorsal CA1 neurons had no effects on R_{in} at RMP and action potential firing following 100 nM corticosterone in the presence of HCN channel blocker (20 μM ZD7288 in the pipette). (A - C) ZD7288 hyperpolarized V_m (A), increased R_{in} at RMP (B), and at -65 mV (C). (D) Representative voltage responses with step current commands ranging from -150 pA to +30 pA (Δ= 20 pA) at RMP before and after bath application of 100 nM corticosterone in the presence of ZD7288. (E and F) 100 nM corticosterone had no effect on V_m (E) and R_{in} at RMP (F) in dorsal CA1 neurons. (G) Representative voltage responses with depolarizing current step (150 pA; 750 ms) at RMP before and after bath application of 100 nM corticosterone in the presence of ZD7288. (E) Action potential firing at -65 mV was not altered in dorsal CA1 neurons. (n=6). Data are expressed as mean ± SEM.

Supplementary Figure S13

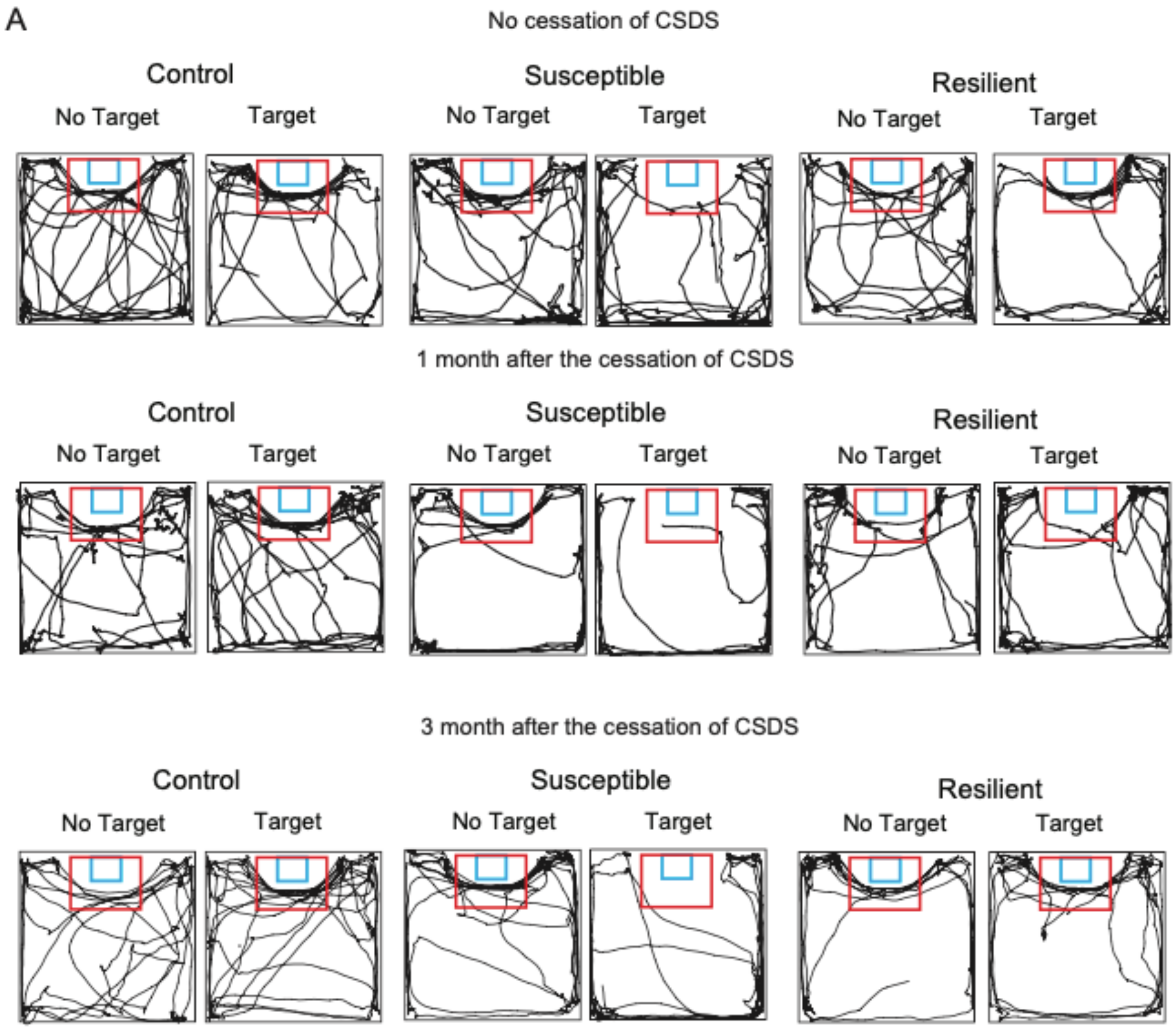

Supplementary Figure S13. Representative video tracking images. (A) Representative video tracking images of control, susceptible, and resilient mice without and with a social target during the social interaction test without the cessation of CSDS, with one month no CSDS, and three months no CSDS.

Supplementary Figure S14

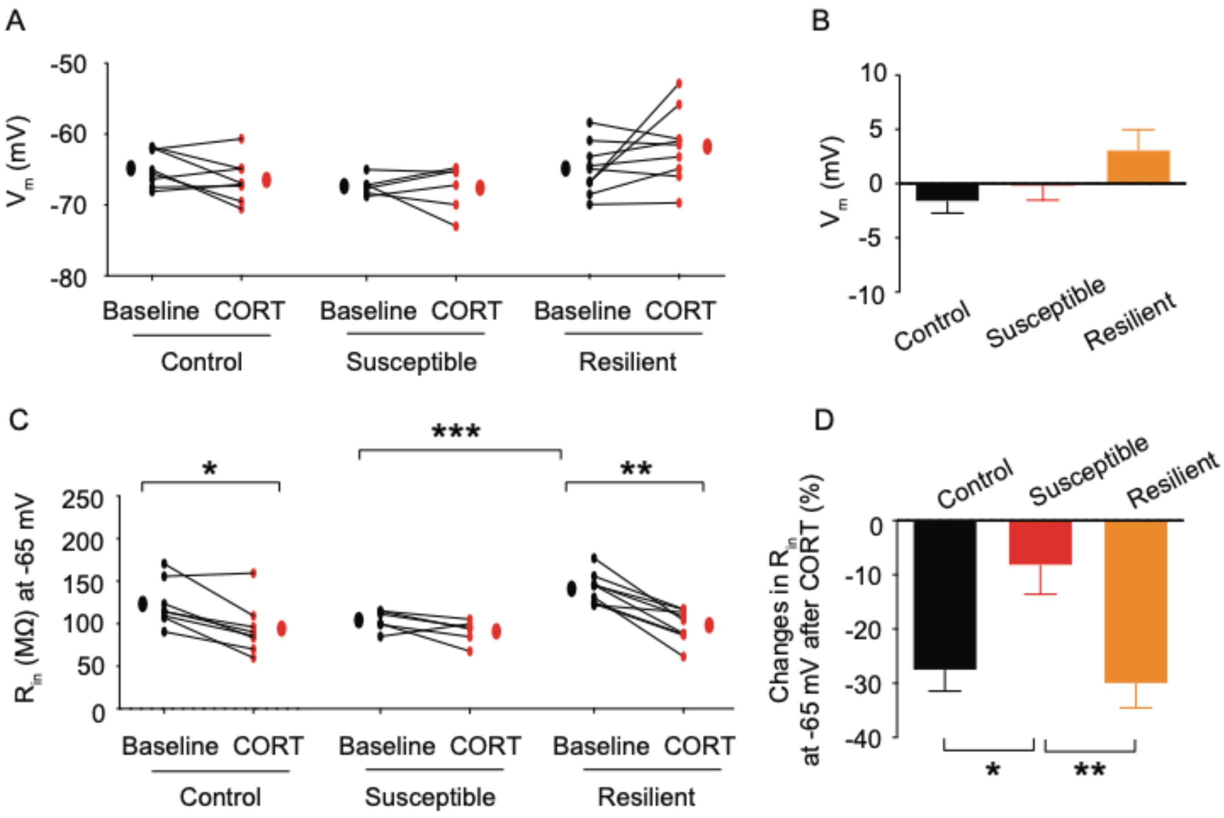

Supplementary Figure S14. Corticosterone (100 nM) reduced R_{in} at -65 mV of the dorsal CA1 neurons in the control and resilient groups, but had no effect on R_{in} in susceptible group. (A and B) Corticosterone had no effect on V_m in dorsal CA1 neurons across groups. (C and D) R_{in} at -65 mV was significantly decreased in dorsal CA1 neurons from the control and resilient mice following corticosterone treatment, but not in susceptible group. Control (n=7), susceptible (n=6), and resilient (n=9). Data are expressed as mean ± SEM.
