# Supplementary material for: Glucocorticoid-glucocorticoid receptor-HCN1 channels reduce neuronal excitability in dorsal hippocampal CA1 neurons
Source: Mol Psychiatry. 2022 Jul 15;27(10):4035–49. doi: 10.1038/s41380-022-01682-9 (PMC9718682; doi:10.1038/s41380-022-01682-9)
Supplement: Supplementary file 2 — Supplementary Table 1 [file 41380_2022_1682_MOESM2_ESM.pdf]

Supplementary Table 1. The properties of a single action potential in dorsal and ventral CA1 neurons across groups.

	Dorsal CA1 neurons			Ventral CA1 neurons		
	Control	Susceptible	Resilient	Control	Susceptible	Resilient
Threshold (mV)	-47.27±0.7	-47.38±1.2	-47.31±1.0	-44.23±1.2	-44.87±1.2	-43.38±1.7
Amplitude (mV)	97.8±4.7	96.7±3.6	99.7±2.7	93.4±3.4	97.1±2.6	97.6±2.1
Half width (ms)	1.95±0.18	1.86±0.09	1.96±0.17	2.05±0.13	2.01±0.17	2.02±0.23
Max dv/dt (mV/ms)	218.2±22.8	216.8±16.4	214.8±9.3	187.4±13.4	184.7±23.5	189.0±22.6
