# Supplementary material for: Glucocorticoid-glucocorticoid receptor-HCN1 channels reduce neuronal excitability in dorsal hippocampal CA1 neurons
Source: Mol Psychiatry. 2022 Jul 15;27(10):4035–49. doi: 10.1038/s41380-022-01682-9 (PMC9718682; doi:10.1038/s41380-022-01682-9)
Supplement: Supplementary file 3 — Statistical Table [file 41380_2022_1682_MOESM3_ESM.pdf]

Supplementary Table 1. Susceptible mice displayed social avoidance and impaired spatial working memory.

Figure 1C			
Group	Control	Susceptible	Resilient
Mean (sec)	35.13	53.51	30.08
S.E.M.	2.609	3.291	3.221
n	39	12	13
One-way ANOVA			
Tukey's multiple comparisons test	Adjusted P Value	ANOVA summary	
Control vs. Susceptible	0.001	F	9.314
Control vs. Resilient	0.5335	P value	0.0003
Susceptible vs. Resilient	0.0005	P value summary	***

Figure 1D			
Group	Control	Susceptible	Resilient
Mean (sec)	76.52	34.07	63.74
S.E.M.	3.757	4.326	5.384
n	39	12	13
One-way ANOVA			
Tukey's multiple comparisons test	Adjusted P Value	ANOVA summary	
Control vs. Susceptible	<0.0001	F	18.45
Control vs. Resilient	0.1533	P value	<0.0001
Susceptible vs. Resilient	0.0025	P value summary	****

Figure 1E			
Group	Control	Susceptible	Resilient
Mean (ratio)	2.524	0.6397	2.327
S.E.M.	0.1616	0.06736	0.2136
n	39	12	13
One-way ANOVA			
Tukey's multiple comparisons test	Adjusted P Value	ANOVA summary	

Control vs. Susceptible	<0.0001	F	22.32
Control vs. Resilient	0.7577	P value	<0.0001
Susceptible vs. Resilient	<0.0001	P value summary	****

Figure 1G			
Group	Control	Susceptible	Resilient
Mean (sec)	36.3	15.01	18.2
S.E.M.	3.928	3.637	2.362
n	33	12	12
One-way ANOVA			
Tukey's multiple comparisons test	Adjusted P Value	ANOVA summary	
Control vs. Susceptible	0.0037	F	7.836
Control vs. Resilient	0.0155	P value	0.001
Susceptible vs. Resilient	0.9079	P value summary	**

Figure 1I			
Group	Control	Susceptible	Resilient
Mean (%)	48.55	39.62	49.38
S.E.M.	2.079	2.283	2.573
n	21	12	13
One-way ANOVA			
Tukey's multiple comparisons test	Adjusted P Value	ANOVA summary	
Control vs. Susceptible	0.0248	F	4.646
Control vs. Resilient	0.9638	P value	0.0149
Susceptible vs. Resilient	0.0269	P value summary	*

Supplementary Table 2. The susceptible group reduced R_{in} and neuronal excitability in dorsal CA1 neurons, but not in ventral CA1 neurons.

Figure 2C			
Group	Control	Susceptible	Resilient
Mean (MΩ)	124.7	82.79	125
S.E.M.	5.641	4.048	6.724
n	9	14	11
One-way ANOVA			
Tukey's multiple comparisons test	Adjusted P Value	ANOVA summary	
Control vs. Susceptible	<0.0001	F	22.08
Control vs. Resilient	0.9993	P value	<0.0001
Susceptible vs. Resilient	<0.0001	P value summary	****

Figure 2E				
Two-way ANOVA				
Source of Variation	% of total variation	P value		
Interaction	2.785	0.045		
Current injection	64.14	<0.0001		
Group	7.95	<0.0001		
The number of action potentials at RMP	Control	Susceptible	Resilient	
N	8	13	10	
	Mean (n)	Mean (n)	Mean (n)	Multiple comparisons test (Tukey)
30 pA	-3.553E-15	0	0	Adjusted P Value
Con vs Res				>0.9999
Con vs Sus				>0.9999
Res vs Sus				>0.9999
60 pA	0.375	0	0.1	
Con vs Res				0.9836
Con vs Sus				0.9663

Res vs Sus				0.9972
90 pA	2.25	0.4615	2.2	
Con vs Res				0.9995
Con vs Sus				0.4606
Res vs Sus				0.4336
120 pA	4.75	1.231	5.2	
Con vs Res				0.9567
Con vs Sus				0.0521
Res vs Sus				0.0142
150 pA	8.375	2.462	8	
Con vs Res				0.9697
Con vs Sus				0.0003
Res vs Sus				0.0003
180 pA	10.25	4	10	
Con vs Res				0.9864
Con vs Sus				0.0001
Res vs Sus				<0.0001
210 pA	12.13	6.385	11.5	
Con vs Res				0.9181
Con vs Sus				0.0005
Res vs Sus				0.001
240 pA	13.63	8.385	13.1	
Con vs Res				0.9415
Con vs Sus				0.0017
Res vs Sus				0.0026
270 pA	14.75	10.46	14	
Con vs Res				0.8843
Con vs Sus				0.0129
Res vs Sus				0.0334
300 pA	16.13	11.85	15.2	
Con vs Res				0.8295
Con vs Sus				0.0132
Res vs Sus				0.0468

Supplementary Table 3. HCN1 protein expression and I_h were significantly increased in dorsal CA1 region/neurons from susceptible group.

Figure 3E			
Group	Control	Susceptible	Resilient
Mean (AU)	1	3.122	1.505
S.E.M.	0.1099	0.5619	0.2247
n	4	4	3
One-way ANOVA			
Tukey's multiple comparisons test	Adjusted P Value	ANOVA summary	
Control vs. Susceptible	0.0083	F	9.142
Control vs. Resilient	0.6503	P value	0.0086
Susceptible vs. Resilient	0.0463	P value summary	**

Figure 3G			
Group	Control	Susceptible	Resilient
Mean (pA)	1.808	8.587	2.547
S.E.M.	0.4358	1.110	0.5641
n	9	11	9
One-way ANOVA			
Tukey's multiple comparisons test	Adjusted P Value	ANOVA summary	
Control vs. Susceptible	<0.0001	F	21.36
Control vs. Resilient	0.8168	P value	<0.0001
Susceptible vs. Resilient	<0.0001	P value summary	****

Figure 3I				
Two-way ANOVA				
Source of Variation	% of total variation	P value		
Interaction	2.722	<0.0001		
V_m	63.51	<0.0001		
Group	6.310	<0.0001		
h current	Control	Susceptible	Resilient	
N	10	22	12	

	Mean (n)	Mean (n)	Mean (n)	Multiple comparisons test (Tukey)
-60 mV	-10.82	-5.063	-5.590	Adjusted P Value
Con vs Sus				0.7167
Con vs Res				0.8038
Sus vs Res				0.9969
-70 mV	-11.17	-12.50	-4.621	
Con vs Sus				0.9824
Con vs Res				0.7102
Sus vs Res				0.4951
-80 mV	-22.17	-29.49	-14.64	
Con vs Sus				0.5836
Con vs Res				0.6369
Sus vs Res				0.0846
-90 mV	-39.45	-62.11	-37.05	
Con vs Sus				0.0067
Con vs Res				0.9550
Sus vs Res				0.0011
-100 mV	-73.78	-96.35	-58.52	
Con vs Sus				0.0069
Con vs Res				0.1592
Sus vs Res				<0.0001
-110 mV	-94.75	-121.0	-78.17	
Con vs Sus				0.0013
Con vs Res				0.1148
Sus vs Res				<0.0001
-120 mV	-103.8	-141.8	-91.76	
Con vs Sus				<0.0001
Con vs Res				0.3182
Sus vs Res				<0.0001
-130 mV	-116.7	-156.6	-101.5	
Con vs Sus				<0.0001
Con vs Res				0.1609
Sus vs Res				<0.0001
-140 mV	-124.7	-168.6	-112.7	
Con vs Sus				<0.0001
Con vs Res				0.3141
Sus vs Res				<0.0001

Figure 3K			
Group	Control	Susceptible	Resilient
Mean (mV)	-101.8	-90.02	-102.1
S.E.M.	2.127	2.668	3.825

n	8	9	8
One-way ANOVA			
Tukey's multiple comparisons test	Adjusted P Value	ANOVA summary	
Control vs. Susceptible	0.0245	F	5.652
Control vs. Resilient	0.9972	P value	0.0104
Susceptible vs. Resilient	0.0208	P value summary	*

Supplementary Table 4. Dorsal CA1 neurons responded to corticosterone more strongly than ventral CA1 neurons.

Figure 4D		
Group	Dorsal CA1	Ventral CA1
Mean (AU)	1.837	1
S.E.M.	0.2418	0.07218
n	5	5
Mann Whitney test		
P value	0.0079	
Exact or approximate P value?	Exact	
P value summary	**	
Significantly different (P < 0.05)?	Yes	
One- or two-tailed P value?	Two-tailed	

Figure 4G		
Group	Baseline	CORT
Mean ((MΩ)	115.3	92.67
S.E.M.	5.694	4.885
n	14	14
Wilcoxon matched-pairs signed rank test		
P value	0.0006	
Exact or approximate P value?	Exact	
P value summary	***	
Significantly different (P < 0.05)?	Yes	
One- or two-tailed P value?	Two-tailed	

Figure 4L			
Two-way ANOVA			
Source of Variation	% of total variation	P value	
Interaction	5.636	<0.0001	
Current injection	50.91	<0.0001	
Group	21.01	<0.0001	

The number of action potentials at RMP	Baseline	CORT	
N	10	10	
	Mean (n)	Mean (n)	Multiple comparisons test (Tukey)
30 pA	0.1	0.1	>0.9999
Baseline vs 100 nM CORT			
60 pA	1.4	0.3	0.998
Baseline vs 100 nM CORT			
90 pA	4.2	0.5	0.1315
Baseline vs 100 nM CORT			
120 pA	7.1	1	0.0006
Baseline vs 100 nM CORT			
150 pA	9.7	2.3	<0.0001
Baseline vs 100 nM CORT			
180 pA	12.1	3.4	<0.0001
Baseline vs 100 nM CORT			
210 pA	13.9	5.5	<0.0001
Baseline vs 100 nM CORT			
240 pA	15.7	7	<0.0001
Baseline vs 100 nM CORT			
270 pA	17	8.5	<0.0001
Baseline vs 100 nM CORT			
300 pA	18.4	9.8	<0.0001
Baseline vs 100 nM CORT			

Figure 4P	Dorsal CA1		
Group	10 nM	100 nM	1000 nM
Mean (%)	-5.574	-22.25	-6.322
S.E.M.	1.869	2.782	3.354
n	3	14	7

Group	Ventral CA1		
Mean (%)	10 nM	100 nM	1uM
S.E.M.	-4.517	-4.27	-3.989
n	1.686	1.85	3.341
	3	8	6
One-way ANOVA	ANOVA summary		
	F	8	
	P value	<0.0001	
	P value summary	****	
Tukey's multiple comparisons test	Adjusted P Value		
Dorsal 10 vs. Dorsal 100	0.0374		
Dorsal 100 vs. Dorsal 1000	0.0028		
Dorsal 100 vs. Ventral 10	0.0229		
Dorsal 100 vs. Ventral 100	0.0003		
Dorsal 100 vs. Ventral 1000	0.001		

Figure 4Q	Dorsal CA1		
Group	10 nM	100 nM	1000 nM
Mean (%)	-2.275	-18.39	-4.919
S.E.M.	3.381	3.1	2.783
n	3	14	7
	Ventral CA1		
Group	10 nM	100 nM	1uM
Mean (%)	-9.492	-9.207	-4.008
S.E.M.	2.407	2.655	3.165
n	3	8	6
One-way ANOVA	ANOVA summary		
	F	3.802	
	P value	0.0074	
	P value summary	**	
Tukey's multiple comparisons test	Adjusted P Value		
Dorsal 100 vs. Dorsal 1000	0.0311		
Dorsal 100 vs. Ventral 1000	0.0281		

Supplementary Table 5. The GR, HCN channels, and the PKA pathway were all involved in corticosterone-induced upregulation of functional I_h.

Figure 5G		
Group	Vehicle	100 nM CORT
Mean (pA)	1.713	9.500
S.E.M.	0.4540	0.9607
n	12	8
Mann Whitney test		
P value	<0.0001	
Exact or approximate P value?	Exact	
P value summary	****	
Significantly different (P < 0.05)?	Yes	
One- or two-tailed P value?	Two-tailed	

Figure 5H		
Group	Baseline	DEX
Mean (MΩ)	122.5	90.31
S.E.M.	8.861	3.779
n	7	7
Mann Whitney test		
P value	0.0175	
Exact or approximate P value?	Exact	
P value summary	*	
Significantly different (P < 0.05)?	Yes	
One- or two-tailed P value?	Two-tailed	

Figure 5H		
Group	Baseline	CORT
Mean ((MΩ)	111.8	84.05
S.E.M.	6.073	1.935
n	5	5
Mann Whitney test		
P value	0.0079	

Exact or approximate P value?	Exact	
P value summary	**	
Significantly different (P < 0.05)?	Yes	
One- or two-tailed P value?	Two-tailed	

Figure 5H		
Group	Baseline	KN-62
Mean ((MΩ)	129.9	101.6
S.E.M.	7.697	3.393
n	7	7
Mann Whitney test		
P value	0.0022	
Exact or approximate P value?	Exact	
P value summary	**	
Significantly different (P < 0.05)?	Yes	
One- or two-tailed P value?	Two-tailed	

Figure 5J		
Group	Baseline	DEX
Mean ((MΩ)	129.5	95.47
S.E.M.	8.743	8.142
n	7	7
Mann Whitney test		
P value	0.0157	
Exact or approximate P value?	Exact	
P value summary	*	
Significantly different (P < 0.05)?	Yes	
One- or two-tailed P value?	Two-tailed	

Figure 5J		
Group	Baseline	CORT
Mean ((MΩ)	114.1	86.81
S.E.M.	4.424	2.888
n	5	5

Mann Whitney test		
P value	0.0079	
Exact or approximate P value?	Exact	
P value summary	**	
Significantly different (P < 0.05)?	Yes	
One- or two-tailed P value?	Two-tailed	

Figure 5J		
Group	Baseline	KN-62
Mean ((MΩ)	140.2	109.9
S.E.M.	12.93	10.36
n	7	7
Mann Whitney test		
P value	0.0175	
Exact or approximate P value?	Exact	
P value summary	*	
Significantly different (P < 0.05)?	Yes	
One- or two-tailed P value?	Two-tailed	

Supplementary Table 6. Susceptible mice re-exposed to novel aggressor mice displayed a persistent social avoidance, which was associated with elevated I_h and was insensitive to corticosterone effects

Figure 6B	No cessation of CSDS		
Group	Control	Susceptible	Resilient
Mean (ratio)	2.705	0.7461	2.116
S.E.M.	0.2822	0.09584	0.3475
n	7	3	4
One-way ANOVA			
Tukey's multiple comparisons test	Adjusted P Value	ANOVA summary	
Control vs. Susceptible	0.0034	F	9.142
Control vs. Resilient	0.3662	P value	0.0046
Susceptible vs. Resilient	0.05	P value summary	**

Figure 6C	1 month after the cessation of CSDS		
Group	Control	Susceptible	Resilient
Mean (ratio)	1.532	0.6201	1.571
S.E.M.	0.2054	0.1235	0.2352
n	7	5	2
One-way ANOVA			
Tukey's multiple comparisons test	Adjusted P Value	ANOVA summary	
Control vs. Susceptible	0.0128	F	6.856
Control vs. Resilient	0.9934	P value	0.0117
Susceptible vs. Resilient	0.0646	P value summary	*

Figure 6D	3 month after the cessation of CSDS		
Group	Control	Susceptible	Resilient
Mean (ratio)	1.823	0.4781	1.986
S.E.M.	0.354	0.08012	0.207

n	7	3	4
One-way ANOVA			
Tukey's multiple comparisons test	Adjusted P Value	ANOVA summary	
Control vs. Susceptible	0.0518	F	4.02
Control vs. Resilient	0.9319	P value	0.0489
Susceptible vs. Resilient	0.0487	P value summary	*

Figure 6F				
Two-way ANOVA				
Source of Variation	% of total variation	P value		
Interaction	3.501	0.002		
Row Factor	57.51	<0.0001		
Column Factor	16.72	<0.0001		
The number of action potentials at RMP	Control	Susceptible	Resilient	
N	8	13	10	
	Mean (n)	Mean (n)	Mean (n)	Multiple comparisons test (Tukey)
30 pA	-3.553E-15	0	0	Adjusted P Value
Con vs Sus				>0.9999
Con vs Res				>0.9999
Sus vs Res				>0.9999
60 pA	0.375	0	0.1	
Con vs Sus				0.9317
Con vs Res				0.3965
Sus vs Res				0.2627
90 pA	2.25	0.4615	2.2	
Con vs Sus				0.3382
Con vs Res				0.1216
Sus vs Res				0.0037
120 pA	4.75	1.231	5.2	
Con vs Sus				0.018
Con vs Res				0.0262

Sus vs Res				<0.0001
150 pA	8.375	2.462	8	
Con vs Sus				0.0045
Con vs Res				0.0063
Sus vs Res				<0.0001
180 pA	10.25	4	10	
Con vs Sus				0.0057
Con vs Res				0.013
Sus vs Res				<0.0001
210 pA	12.13	6.385	11.5	
Con vs Sus				0.0033
Con vs Res				0.0244
Sus vs Res				<0.0001
240 pA	13.63	8.385	13.1	
Con vs Sus				0.0039
Con vs Res				0.0282
Sus vs Res				<0.0001
270 pA	14.75	10.46	14	
Con vs Sus				0.0039
Con vs Res				0.031
Sus vs Res				<0.0001
300 pA	16.13	11.85	15.2	
Con vs Sus				0.0007
Con vs Res				0.0746
Sus vs Res				<0.0001

Figure 6H			
Group	Control	Susceptible	Resilient
Mean (pA)	2.34	8.296	3.29
S.E.M.	0.467	1.538	0.6471
n	8	5	5
One-way ANOVA			
Tukey's multiple comparisons test	Adjusted P Value	ANOVA summary	
Control vs. Susceptible	0.0005	F	12.77
Control vs. Resilient	0.7186	P value	0.0006
Susceptible vs. Resilient	0.0055	P value summary	***

Figure 6M	Control group	
Group	Baseline	CORT
Mean ((MΩ)	131.5	92.19

S.E.M.	10.15	8.929
n	8	8
Wilcoxon matched-pairs signed rank test		
P value	0.0078	
Exact or approximate P value?	Exact	
P value summary	**	
Significantly different (P < 0.05)?	Yes	
One- or two-tailed P value?	Two-tailed	

Figure 6M	Susceptible group	
Group	Baseline	CORT
Mean ((MΩ)	99.1	90.45
S.E.M.	3.326	4.354
n	6	6
Wilcoxon matched-pairs signed rank test		
P value	0.1562	
Exact or approximate P value?	Exact	
P value summary	ns	
Significantly different (P < 0.05)?	No	
One- or two-tailed P value?	Two-tailed	

Figure 6M	Resilient group	
Group	Baseline	CORT
Mean ((MΩ)	144.2	108.3
S.E.M.	5.252	5.429
n	9	9
Wilcoxon matched-pairs signed rank test		
P value	0.0039	
Exact or approximate P value?	Exact	
P value summary	**	
Significantly different (P < 0.05)?	Yes	
One- or two-tailed P value?	Two-tailed	

Figure 6M	Before CORT treatment		
Group	Control	Susceptible	Resilient
Mean ((MΩ)	131.5	99.1	144.2
S.E.M.	10.15	3.326	5.252
n	8	6	9
One-way ANOVA			
Tukey's multiple comparisons test	Adjusted P Value	ANOVA summary	
Control vs. Susceptible	0.0194	F	9.24
Control vs. Resilient	0.4094	P value	0.0014
Susceptible vs. Resilient	0.0011	P value summary	**

Figure 6N	After CORT treatment		
Group	Control	Susceptible	Resilient
Mean ((%)	-29.98	-7.967	-24.68
S.E.M.	3.584	2.142	3.204
n	8	6	9
One-way ANOVA			
Tukey's multiple comparisons test	Adjusted P Value	ANOVA summary	
Control vs. Susceptible	0.0005	F	11
Control vs. Resilient	0.4549	P value	0.0006
Susceptible vs. Resilient	0.0054	P value summary	***
